# Supplementary material for: High kinetic inductance cavity arrays for compact band engineering and topology-based disorder meters
Source: Nat Commun. 2025 Apr 16;16:3396. doi: 10.1038/s41467-025-58595-8 (PMC12003849; doi:10.1038/s41467-025-58595-8)
Supplement: Supplementary file 1 — Supplementary Information [file 41467_2025_58595_MOESM1_ESM.pdf]

Supplementary Material
High kinetic inductance cavity arrays for compact band engineering and topology-based disorder meters

Vincent Jouanny,[1,2,*] Simone Frasca,[1,2] Vera Jo Weibel,[1,2] Léo Peyruchat,[1,2] Marco Scigliuzzo,[2,3] Fabian Oppliger,[1,2] Franco De Palma,[1,2] Davide Sbroggiò,[1,2] Guillaume Beaulieu,[1,2] Oded Zilberberg,[4] and Pasquale Scarlino[1,2,†]

[1] *Hybrid Quantum Circuits Laboratory (HQC), Institute of Physics, École Polytechnique Fédérale de Lausanne (EPFL), 1015, Lausanne, Switzerland*
[2] *Center for Quantum Science and Engineering, Institute of Physics, École Polytechnique Fédérale de Lausanne (EPFL), 1015, Lausanne, Switzerland*
[3] *Laboratory of Photonics and Quantum Measurements (LPQM), Institute of Physics, École Polytechnique Fédérale de Lausanne (EPFL), 1015, Lausanne, Switzerland*
[4] *Department of Physics, University of Konstanz, D-78457 Konstanz, Germany*

(Dated: February 26, 2025)

Appendix A: Supplementary figures

Appendix B: Model of CCAs with $M = 1$

In this section, we perform approximations on the derivation of the Hamiltonian presented in Methods Sec. VI, which allows the implementation of an initial simplified tight-binding interpretation (only including first nearest neighbor interaction) of the CCAs. We then enrich the model to include higher-neighbor coupling terms and study their effect on the band structure.

From the model developed in Methods, we start with a CCA with N resonators with $M = 1$, assuming a CCA with constant coupling and resonant frequency. We define the capacitance to ground as C_g, the inductance to ground as L_g, and the mutual capacitances between resonators as C_1. We proceed to two assumptions on the capacitance matrix developed in Methods (Eq. (12))). The first one is to neglect the stray capacitance between resonators higher than their first neighbors, $C_{i,i+j} = 0$ for $j > 1$. The second assumption has to do with the inversion of the capacitance matrix, where the terms $C_{i,i+j}C_{i+j,i}$ are neglected for orders higher than 1, valid when $C_{i,i+1}/C_{\Sigma,i}$ is small as mentioned in the main text. Doing so, the inverse of the capacitance matrix becomes

$$\left[C^{-1}\right] = L_g\omega_r^2 \begin{pmatrix} 1 & \beta & 0 & \dots & \dots & 0 \\ \beta & 1 & \beta & \ddots & \ddots & \vdots \\ 0 & \beta & 1 & \beta & \ddots & \vdots \\ \vdots & \ddots & \ddots & \ddots & \ddots & \vdots \\ \vdots & \ddots & \ddots & \ddots & \ddots & \beta \\ 0 & \dots & \ddots & \ddots & \beta & 1 \end{pmatrix}, \tag{B1}$$

where we have

$$\beta = \frac{C_1}{C_\Sigma}, \tag{B2}$$

$$\omega_r = \frac{1}{\sqrt{L_g C_\Sigma}}. \tag{B3}$$

* vincent.jouanny@epfl.ch
† pasquale.scarlino@epfl.ch

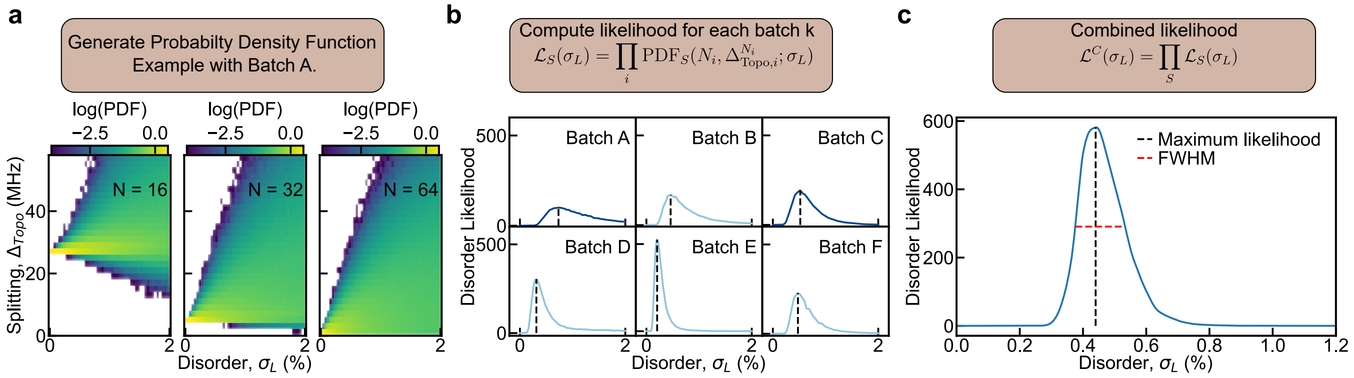

Supplementary Figure S1. **Likelihood analysis. a.** Probability density functions of batch A as a function of the disorder, σ_L, and the splitting between the SSH edge modes, Δ^N_{Topo}, for different number of cavities, N. **b.** Likelihood functions computed for each batch. The coupling configuration of each batch is represented with the same color code as in Fig. **??** of the main text. The maximum likelihood of each batch is shown with a black dashed lines. **c.** Combined likelihood between all batches. The maximum likelihood is indicated with a black dashed line, the FWHM is indicated with the red dashed line.

C_Σ is the total capacitance of the resonator defined as $C_\Sigma = C_g + 2C_1$. With this simplified capacitance matrix, the Hamiltonian can now be written using Eq. (16) of Methods as

$$\begin{aligned} H =& \frac{1}{2}\sum_{n=1}^{N}\left(L_g\omega_r^2 Q_n + \frac{1}{L_g}\phi_n\right) \\ &+ \frac{L_g\omega_r^2\beta}{2}\sum_{n=1}^{N-1}\left(Q_nQ_{n+1} + Q_{n+1}Q_n\right). \end{aligned} \tag{B4}$$

We now quantize this Hamiltonian, assuming the commutation relation $\left[\hat{Q}_n, \hat{\phi}_m\right] = i\hbar\delta_{nm}$ [1] which allow to define,

$$\hat{Q}_n = \sqrt{\frac{\hbar}{2L_g\omega_r}}\left(\hat{a}_n^\dagger + \hat{a}_n\right), \tag{B5}$$

$$\hat{\phi}_n = \sqrt{\frac{\hbar L_g\omega_r}{2}}\left(\hat{a}_n^\dagger - \hat{a}_n\right), \tag{B6}$$

where $\hat{a}_n^\dagger$ $(\hat{a}_n)$, is the annihilation (creation) operators at site n. Including the above relations Eq. (B5) and Eq. (B6) in the Hamiltonian, Eq. (B4), we get

$$\begin{aligned} \hat{H} =& \sum_{n=1}^{N}\hbar\omega_r\left(\hat{a}_n^\dagger\hat{a}_n + \frac{1}{2}\right) \\ &+ \hbar J\sum_{n=1}^{N-1}\left(\hat{a}_n^\dagger\hat{a}_{n+1} + h.c.\right), \end{aligned} \tag{B7}$$

where

$$J = \frac{\omega_r}{2}\frac{C_1}{C_\Sigma}, \tag{B8}$$

is the coupling between the cavities. In this case, the tight-binding model predicts the emergence of a passband centered around the bare resonance frequency of a single cavity ($\omega_r = 1/\sqrt{L_g(C_g + 2C_1)}$) with a span of $4J$ with N modes.

Now, we investigate how coupling terms above first neighbor modify this simplified model. Higher order coupling terms will generate terms of type

$$\hat{K}_q = \sum_{n=1}^{N-q}\hbar J^{(q)}\left(\hat{a}_n^\dagger\hat{a}_{n+q} + h.c.\right), \tag{B9}$$

where q is the order of the coupling term. $J^{(q)}$ represents coupling terms to the qth nearest neighbor cavity. In the following, we refer to terms of order $q = 2$, 3 and 4 as J', J'' and J''', respectively. These higher coupling terms will simply add up to the real space Hamiltonian Eq. (B7) as

$$\hat{H}_{\mathrm{tot}} = \hat{H} + \sum_{i=2}^{I}\hat{K}_i, \tag{B10}$$

up to the Ith order. These higher coupling terms can arise due to the two origin mentioned above, meaning higher order terms due to the inversion of the capacitance matrix and direct stray mutual capacitance. In Fig. S2, we show the scaling of these effects on the next nearest neighbor coupling using the Hamiltonian introduced in the Methods section.

In Fig. S2**c**, we show the effect of the increase of the relative coupling capacitance with respect to the total capacitance, C_1/C_Σ, on the relative higher m^{th} order photonic hopping rates, $J^{(m)}/J_1$, when the stray mutual capacitance, $C' = C_{i,i+2}$, is set to 0. As the relative coupling capacitance is increased, higher-order terms become more important. In the figure, we highlight the typical coupling ratios, C_1/C_Σ extracted for the rectangular (6%) and hexagonal (26 %) CCA. The higher neighbor coupling terms in the hexagonal CCA mainly arise due to a high ratio C_1/C_Σ. In Fig. S2**d**, we show the effect of

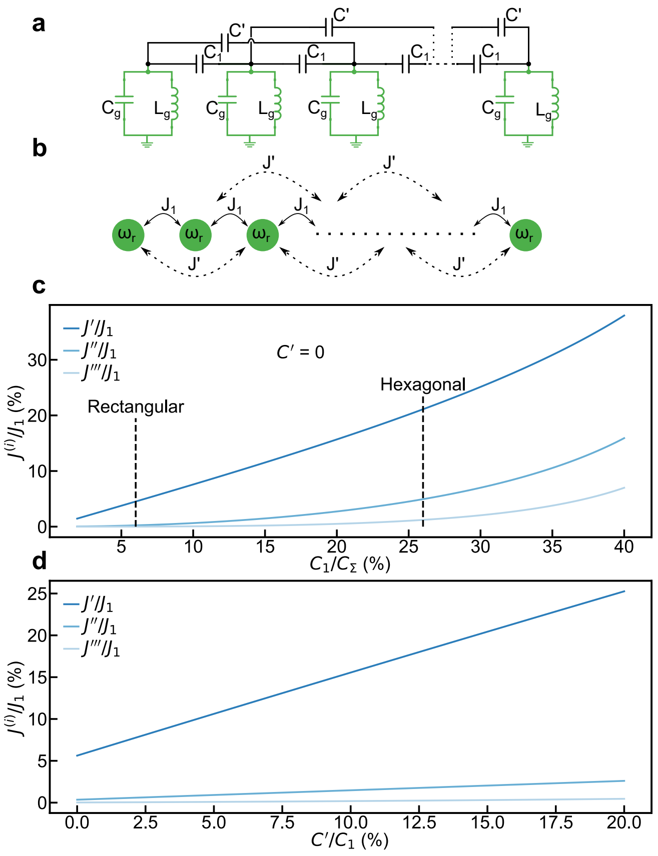

Supplementary Figure S2. **Simulation of higher order coupling terms. a.** Circuit schematic of the CCA under consideration. The resonators, highlighted in green, with capacitance C_g and inductance L_g to ground, respectively, are coupled to the first nearest neighbor (NN) with the capacitances C_1. We represent the second NN coupling capacitance with C'. **b.** Equivalent CCA schematic. The resonators are represented as cavities with resonant frequency $\omega_r/2\pi$. The first and second NN coupling are respectively indicated with J_1 and J'. **c.** Coupling ratio $J^{(i)}/J_1$ of the coupling to the ith NN over C_1/C_Σ. The evolution of second NN coupling J', third NN coupling J'' and fourth NN coupling J''' are reported. The two dashed lines indicate the typical C_1/C_Σ for the rectangular (Fig. 1**b**) and hexagonal designs (Fig. 1**d**). **d.** $J^{(i)}/J_1$ as a function of C'/C_1. The simulations in panel **c** and **d** are performed using Eq. (18) in Methods Sec. VI.

the relative stray capacitance, C'/C_1, on $J^{(m)}/J_1$. J'/J_1 increases linearly, as expected from Eq. (B8). Additionally, we see that as C' increases, higher and lower order coupling terms also start to emerge.

In summary, for the rectangular CCA geometry, the second nearest neighbour couplings $J' \approx 10\% J_1$ (dominated by direct stray capacitive coupling), while for the hexagonal one, $J' \approx 22\% J_1$ (due to high C_1/C_Σ ratio). It is worth remarking that a tight-binding Hamiltonian, which includes only the first two terms in Eq. (B10), represents a valid approximation only when the ratio J'/J_1 is negligible.

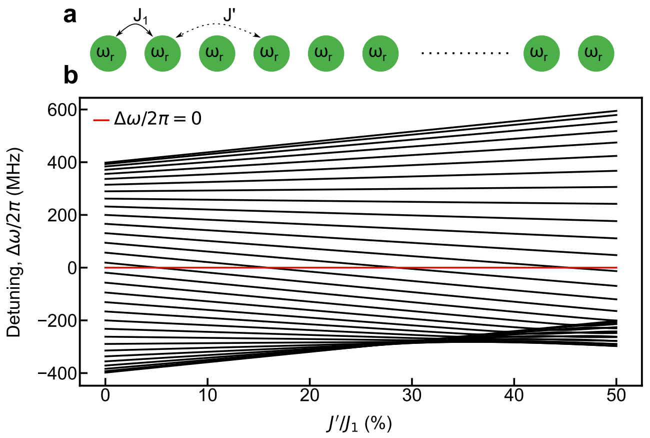

Supplementary Figure S3. **Simulation of the influence of second nearest neighbor coupling. a.** Schematic of a uniform CCA with cavity resonant frequency $\omega_r/2\pi$, coupling to first nearest neighbor (NN) J_1 and coupling to second NN J'. **b.** Eigenvalues for a CCA with $N = 32$ and couplings typical of a rectangular geometry, calculated according to Eq. (B10) as a function of J'/J_1. The modes frequencies are represented as a function of the detuning $\Delta\omega = \omega - \omega_r$. The red line represents $\omega = \omega_r$.

Applying periodic boundary conditions to Eq. (B10), we can write this Hamiltonian in momentum space (k-space), using the Fourier transform of the real space creation operator at site n,

$$\hat{a}_n = \frac{1}{\sqrt{N}} \sum_k e^{jknd} \hat{a}_k, \tag{B11}$$

where d is the size of the unit cell and k is the wavevector. From the equation above we find the k-space Hamiltonian,

$$\begin{aligned} H(k) = \sum_k \hbar \left(\omega_r + 2J' \cos\left(2kd\right)\right) \hat{a}_k^\dagger \hat{a}_k \\ + 2\hbar J \hat{a}_k^\dagger \hat{a}_{k+1} \cos\left(kd\right). \end{aligned} \tag{B12}$$

The corresponding dispersion relation reads,

$$\omega_k = \omega_r + 2J_1 \cos\left(kd\right) + 2J' \cos\left(2kd\right). \tag{B13}$$

In the limit where $J' = 0$, we find the standard cosine dispersion relation of the tight-binding model, spanned over $4J$ and centered around ω_r. However, when $J' \neq 0$, significant deviations between the tight-binding model spectrum and this model become evident. It manifests in an asymmetric passband with respect to ω_r, resulting in higher mode density at lower frequencies and the eigenmodes of the CCA shift upward with respect to ω_r (Fig. S3). The effect on the dissipations in the passband is studied in App. E.

Appendix C: Gapped CCAs with $M \geq 2$

In this section, we model CCAs with gapped spectrum and study, as in the previous section, how they are affected by higher neighbor coupling terms. In order to open bandgaps, several methods are possible relying on the generation of unit cells [2, 3]. Depending on the coupling configuration inside the unit cell, the number of cavities per unit cell will define the number of bandgaps: for M cavities per unit cell, there will be $M-1$ bandgaps.

We first show the case for a dimerized CCA ($M=2$), which is defined by alternating the mutual capacitances C_1 and C_2, while keeping the same inductance to ground, L_g, and constant resonance frequency among the resonators(see Sec. II of the main text). In this case, the inverse of the capacitance matrix (Eq. (12)) can be rewritten as,

$$\left[C^{-1}\right] = L_g\omega_r^2 \begin{pmatrix} 1 & \beta_1 & 0 & \dots & \dots & 0 \\ \beta_1 & 1 & \beta_2 & \ddots & \ddots & \vdots \\ 0 & \beta_2 & 1 & \beta_1 & \ddots & \vdots \\ \vdots & \ddots & \ddots & \ddots & \ddots & \vdots \\ \vdots & \ddots & \ddots & \ddots & \ddots & \beta_1 \\ 0 & \dots & \ddots & \ddots & \beta_1 & 1 \end{pmatrix}, \tag{C1}$$

where the stray next nearest neighbor coupling capacitances and the higher order terms in C_1C_2, C_1^2, C_2^2 have been neglected, as for Eq. (B1) in App. B, and

$$\beta_i = C_i/C_\Sigma. \tag{C2}$$

We can then rewrite the Hamiltonian using Eq. (16) as

$$\begin{aligned} H =& \frac{1}{2}\sum_{n=1}^{N} L_g\omega_r^2 \left[\left(Q_n^A\right)^2 + \left(Q_n^B\right)^2\right] \\ &+ \frac{1}{2}\sum_{n=1}^{N}\frac{1}{L_g}\left[\left(\phi_n^A\right)^2 + \left(\phi_n^B\right)^2\right] \\ &+ \frac{L_g\omega_r^2\beta_1}{2}\sum_{n=1}^{N-1}\left(Q_n^A Q_n^B + Q_n^A Q_n^B\right) \\ &+ \frac{L_g\omega_r^2\beta_2}{2}\sum_{n=1}^{N-1}\left(Q_n^A Q_{n+1}^B + Q_{n+1}^A Q_n^B\right). \end{aligned} \tag{C3}$$

We quantize this Hamiltonian by introducing the quantized charge, $\hat{Q}_n^S$, and flux, $\hat{\phi}_n^S$, operators, acting on the Sth sub-lattice site of the nth unit cell, satisfying the commutation relation,

$$\left[\hat{Q}_n^{S'}, \hat{\phi}_m^S\right] = i\hbar\delta_{n,m}\delta_{S,S'}. \tag{C4}$$

They are defined as,

$$\hat{Q}_n^S = \sqrt{\frac{\hbar}{2L_g\omega_r}}\left(\hat{s}_n^\dagger + \hat{s}_n\right) \tag{C5}$$

$$\hat{\phi}_n^S = \sqrt{\frac{\hbar L_g\omega_r}{2}}\left(\hat{s}_n^\dagger - \hat{s}_n\right), \tag{C6}$$

where $\hat{s}_n^\dagger$ ($\hat{s}_n$) is the annihilation (creation) operator of sub-lattice S of the nth unit cell.
Inserting Eq. (C5) and Eq. (C6) into Eq. (C3), we find the dimerized Hamiltonian,

$$\begin{aligned} \hat{H} =& \hbar\omega_r\sum_{n=1}^{N}\left(\hat{a}_n^\dagger\hat{a}_n + \hat{b}_n^\dagger\hat{b}_n\right) \\ &+ \underbrace{\hbar J_1\sum_{n=1}^{N}\left(\hat{a}_n^\dagger\hat{b}_n + \hat{a}_n\hat{b}_n^\dagger\right)}_{\text{Intracell coupling}} \\ &+ \underbrace{\hbar J_2\sum_{n=1}^{N-1}\left(\hat{a}_{n+1}^\dagger\hat{b}_n + \hat{a}_{n+1}\hat{b}_n\right)}_{\text{Intercell coupling}}. \end{aligned} \tag{C7}$$

A higher neighbor coupling term can be added similarly to the approach in App. B.

Analogously to the procedure described in App. B, one can derive the k-space Hamiltonian to get a grasp of the effect of second neighbor coupling to the mode structure. To do so, we use the Fourier transform of the normal space field operator, for both A and B sublattice, as in Eq. (B11). We find the following Hamiltonian in reciprocal space,

$$\begin{aligned} \hat{H}(k) = \hbar\sum_k \Big[&\left(\omega_0 + 2J'\cos(kd)\right)\left(\hat{a}_k^\dagger\hat{a}_k + \hat{b}_k^\dagger\hat{b}_k\right) \\ &\left(J_1 + J_2e^{-ikd}\right)\hat{a}_k^\dagger\hat{b}_k + \left(J_1 + J_2e^{ikd}\right)\hat{a}_k\hat{b}_k^\dagger\Big]. \end{aligned} \tag{C8}$$

By diagonalizing the above Hamiltonian, we recover the dispersion relation,

$$\omega_\pm/\hbar = \omega_0 + 2J'\cos(kd) \pm \sqrt{J_1^2 + J_2^2 + 2J_1J_2\cos(kd)}. \tag{C9}$$

Here again, we can see that J' will have a k-dependent effect on the band dispersion. We simulate its effect on the mode distribution in Fig. S4**b**. Increasing second neighbor coupling compresses the lower band and dilates the upper band. This effect can create strong band asymmetry and could be a way to engineer extremely high-density bands.

One can generalize the model previously introduced for multiple gap systems ($M > 1$), where we follow the same

recipe as before:

$$\begin{aligned}\hat{H} &= \hbar\omega_r \sum_{n=1}^{N/M}\sum_{m}^{M} \hat{a}^{\dagger}_{m,n}\hat{a}_{m,n} \\ &+\hbar \sum_{n=1}^{N/M}\sum_{m=1}^{M-1} J_{m,m+1}\left(\hat{a}^{\dagger}_{m,n}\hat{a}_{m+1,n} + h.c.\right) \\ &+\hbar \sum_{n=1}^{N/M-1} J_{M,1}\left(\hat{a}^{\dagger}_{M,n}\hat{a}_{1,n+1} + h.c.\right),\end{aligned} \tag{C10}$$

where $\hat{a}_{m,n}$ is the creation operator on the mth cavity in the nth unit cell.

Increasing second neighbor coupling will have the same effect as for the case of single gap devices: compression (expansion) of the lower (upper) bandgaps (Fig. S4**d**).

In the measurements presented in Fig. 2**e** of the main text, we see a clear asymmetry in the bandgap size of the spectra with more than 1 gap. This asymmetry can be explained by a systematic deviation of the resonator's inductances of the real device with respect to the designed one. We attribute this deviation to the resonant frequency difference between the resonators at the edge and in the bulk of the unit cells. In the design, this effect was taken into account by having different inductances for edge and bulk resonators of a unit cell (see App. D). This effect is highlighted in Fig. S5, where we show that errors on the estimation of the inductance as low as 0.6 nH, about 1.5% of the total inductance, drastically affects the band structure.

Appendix D: Design and simulation

All designs were created in the *.gds* format using the gdspy Python library. To ensure the *lumpedness* of the designed resonators, the inductor and capacitor self-resonances' are designed to be much higher than the frequency range we are working in. The typical design workflow begins with an initial tight-binding simulation based on the model established in App. B and App. C. Subsequently, we use Sonnet simulation software to simulate the frequency response of the designs. In this simulation, the inductance is set by defining the number of squares in the inductor to match the required inductance corresponding to the kinetic inductance of the film. From this simulation, we extract the impedance and resonant frequencies of the design.

For the rectangular CCAs, the calibration of the coupling rate between resonators is achieved by simulating two resonators positioned adjacent to each other, with a distance denoted as d_r (d_p) with respect to the neighboring resonator (microwave feedline), as depicted in Fig. S6**a**.

The coupling between two resonators is proportional to $J_{i,i+1} \propto \frac{C_{i,i+1}}{C_\Sigma}$, with $C_{i,i+1}$ being the coupling capacitance and C_Σ the total capacitance of the resonator. As the distance between the two resonators is increased, the coupling between them is reduced as C_c is decreasing (Fig. S6**b**).

For the hexagonal CCAs, the calibration of the coupling rate between resonators is implemented by sweeping the width of the coupling capacitor C_w from 15 to 35 µm for three different finger width equal to the finger spacing $f_w = 1, 2, 3$ µm (see Fig. S7). We then extract the mode splitting from which we infer the coupling between the cavities.

For $M = 1$ cavities per unit cell, all cavities are coupled to one another with the same mutual capacitance, $C_{i,i+1} = C_1$, have a capacitance to ground, C_g, and an inductance to ground, L_g. In this case, all cavities will have the same total capacitance, i.e. $C_\Sigma = Cg + 2C_1$. This condition ensures that all cavities have the same resonant frequency, $\omega_r = 1/\sqrt{L_g C_\Sigma}$. Maintaining a constant resonant frequency among all the resonators in the array poses a challenge due to the presence of local fabrication imperfections and edge coupling points to the input-output ports. While the former is determined by fabrication capabilities, the latter can be mitigated by introducing "ghost" cavities, as indicated in blue in the SEM micrographs (Figs. 1**b** and **d** of the main text). Such elements are designed to mimic the capacitive environment of bulk resonators, ensuring uniform resonant frequencies also for the 1st and Nth resonators of the CCA, provided that all resonators share identical inductance values. These "ghost" elements are designed to be non-resonant with the CCA and are electrically connected to the input/output waveguide, serving as input/output ports of the CCA with capacitance $C_p \approx C_1$, C_p being the capacitor between the coupling ports and the edge cavities.

For $M = 2$, the cavities are coupled in an alternative fashion with the capacitances C_1 and C_2, and have a capacitance to ground, C_g, and an inductance to ground, L_g. Similarly to the case with $M = 1$, the cavities will all have the same total capacitance, i.e. $C_\Sigma = C_g + C_1 + C_2$ for each cavity. Hence, they will have the same resonant frequency, $\omega_r/2\pi$, provided that the coupling capacitance to the input/output waveguides is equal to C_2.

In the dimer case ($M = 2$), each resonator presents the same two coupling capacitances, in an alternating fashion, which automatically satisfies the resonant condition. However, for $M > 2$, the translation uniformity for a single resonator is not preserved anymore. Therefore, accomplishing the resonant frequency condition for all cavities requires precise control over the mutual and ground capacitances. We address this challenge by utilizing RF simulation software to calibrate the resonant frequency as a function of the coupling strength of each of the cavities in the unit cell. In fact, for $M > 2$, the cavities in the unit cell are coupled to one another with $C_1, C_2, C_3, \ldots, C_{M-1}$ and with C_M between unit cells. They have a capacitance to ground, $C_{1g}, C_{2g}, C_{3g}, \ldots, C_{Mg}$ and inductance to ground $L_{1g}, L_{2g}, L_{3g}, \ldots, L_{Mg}$. In this case the cavities within the unit cell will each have a different total

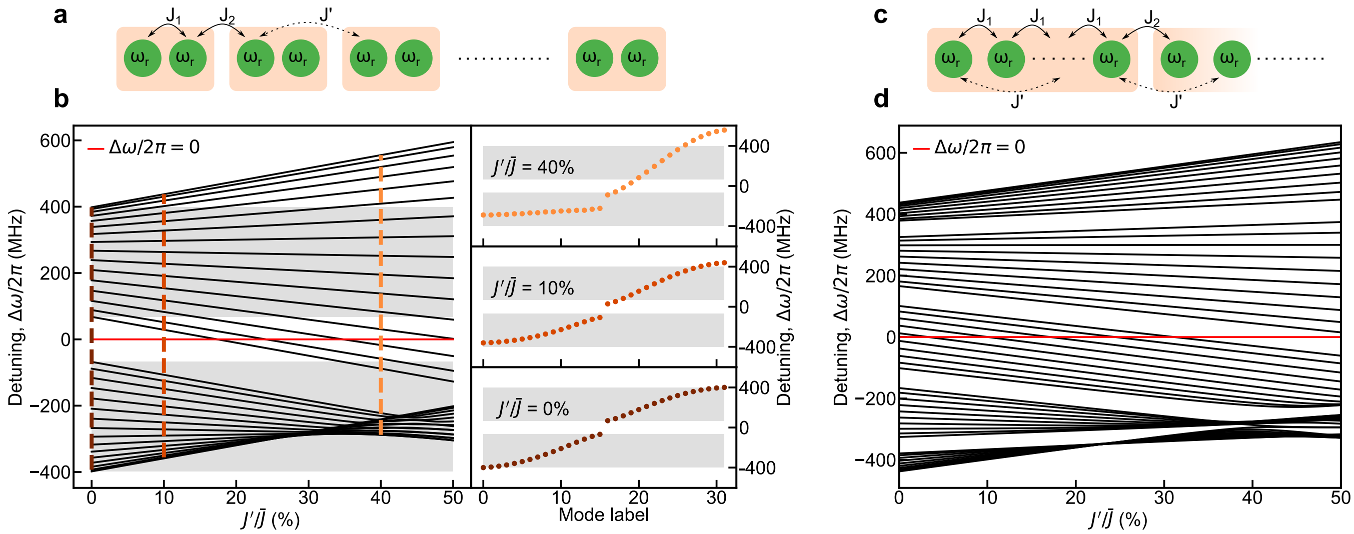

Supplementary Figure S4. **Simulation of the influence of second neighbor coupling on gapped CCAs. a.** Schematic of a dimerized ($M = 2$) CCA with cavity resonant frequency $\omega_r/2\pi$, intracell coupling J_1, intercell coupling J_2 and coupling to second nearest neighbor (NN) J'. **b.** Left: Eigenvalues for a CCA with $N = 32$ and couplings typical of a rectangular geometry, calculated according to Eq. (C7) as a function of $J'/\bar{J}$, $\bar{J} = 1/2(J_1 + J_2)$. The modes frequencies are represented as a function of the detuning $\Delta\omega = \omega - \omega_r$. The red line represents $\omega = \omega_r$. The grey areas highlight the passbands at $J'/\bar{J} = 0$. Right: Cut of the plot in the left panel with $J'/\bar{J} = 0\%$, 10% and 40%. **c.** Schematic of a multigap ($M > 2$) CCA with cavity resonant frequency $\omega_r/2\pi$, intracell couplings J_1, intercell coupling J_2 and coupling to second nearest neighbor (NN) J'. **d.** Eigenvalues for a CCA with $N = 50$ and couplings typical of a rectangular geometry, calculated according to Eq. (C10) as a function of $J'/\bar{J}$, $\bar{J} = 1/5(4J_1 + J_2)$. The modes frequencies are represented as a function of the detuning $\Delta\omega = \omega - \omega_r$. The red line represents $\omega = \omega_r$.

capacitance, $C_{\Sigma,i} = C_{ig} + C_{i-1} + C_{i+1}$. To ensure the same resonant frequency for all cavities, the inductances have to be adjusted such that, $C_{\Sigma,i}L_i = C_{\Sigma,j}L_j$. In order to simplify the design, the CCAs presented in Fig. 2**e**, are implemented restricting the mutual capacitances to C_1 and C_2, the capacitances to ground to C_{1g} and C_{2g} and the inductances to ground to L_{1g} and L_{2g}.

For instance, for the CCA with $M = 3$ presented in the main text, the unit cell coupling capacitance $\vec{C_c} = (C_1, C_1, C_2)$. Hence, here the cavities at the edges of the unit cell will experience a different capacitive environment than the cavities in the bulk of the CCA. Therefore, we need to adapt the inductances for only two types of cavities: the ones at the edges and the ones in the bulk of the unit cell. $\vec{L_g} = (L_{1g}, L_{2g})$ needs to be adjusted to keep a constant resonant frequency.

The designs of the CCA with multiple gaps were chosen by testing different coupling configurations. The simulations were performed using the Hamiltonian introduced in Eq. (C10) in App. C. The simulated mode profiles of each chosen design measured in Fig. 2**e** are displayed in Fig. S9.

Appendix E: Dissipations

1. Modelling of the CCA dissipations

In this section, we focus on the dissipations occurring in the CCA. We derive a non-Hermitian Hamiltonian and the scattering matrix for the CCA that we implemented. The presence of non-negligible second neighbor coupling among cavities suggests the possibility of coupling between the input waveguide and the second closest resonator to the microwave ports. In our subsequent analysis, we incorporate these couplings and observe their effect, noting that they introduce asymmetry in dissipation with respect to frequency detuning. To model dissipations, we start from the Heisenberg-Langevin equation of motion [4],

$$\begin{aligned}\frac{\partial}{\partial t}\hat{a}_n(t) &= j\left[\hat{H}, \hat{a}_n(t)\right] + \frac{\kappa_n}{2}\hat{a}_n(t) \\ &+ \sqrt{\kappa_{\text{ext}}}\left(\delta_{n,1}\hat{a}_{\text{in,L}}(t) + \delta_{n,N}\hat{a}_{\text{in,R}}(t)\right) \\ &+ \sqrt{\kappa'_{\text{ext}}}\left(\delta_{n,2}\hat{a}_{\text{in,L}}(t) + \delta_{n,N-1}\hat{a}_{\text{in,R}}(t)\right),\end{aligned} \tag{E1}$$

where κ_{ext} and κ'_{ext} are the dissipation rates to the coupling ports from the closest and second closest resonator to the microwave ports, respectively. $\kappa_n = \kappa_{\text{int}} + (\delta_{n,1} + \delta_{n,N})\kappa_{\text{ext}} + (\delta_{n,2} + \delta_{n,N-1})\kappa'_{\text{ext}}$ is the total dissipation of the nth cavity, where κ_{int} is the cavity's internal dissipation. $\hat{a}_{\text{in,L(R)}}$ is the input field on the left (right)

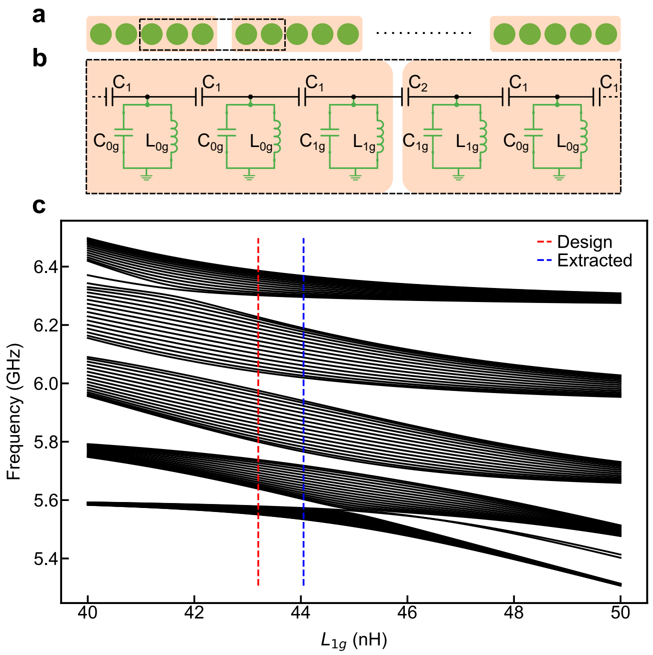

Supplementary Figure S5. **Simulation of the influence of systematic inductance deviation in gapped CCA spectra.** **a.** Schematic of a multigap ($M = 5$) CCA. **b.** Schematic of the lumped-element model of the CCA enclosed by the dashed rectangle in **a**. The mutual coupling capacitance between the resonators inside the unit cell is C_1 and the capacitance between the unit cells is C_2. The capacitance and inductance to ground for the resonators inside the bulk of the unit cell are C_{0g} and L_{0g}, whereas on the edge of the unit cell they are defined as C_{1g} and L_{1g}, respectively. **c.** Simulation of the modes' frequencies as a function of L_{1g}. The red and blue dashed lines highlight the designed and extracted frequency, respectively. The simulation in panel **c** is performed using the Hamiltonian Eq. (16) in Methods Sec. VI

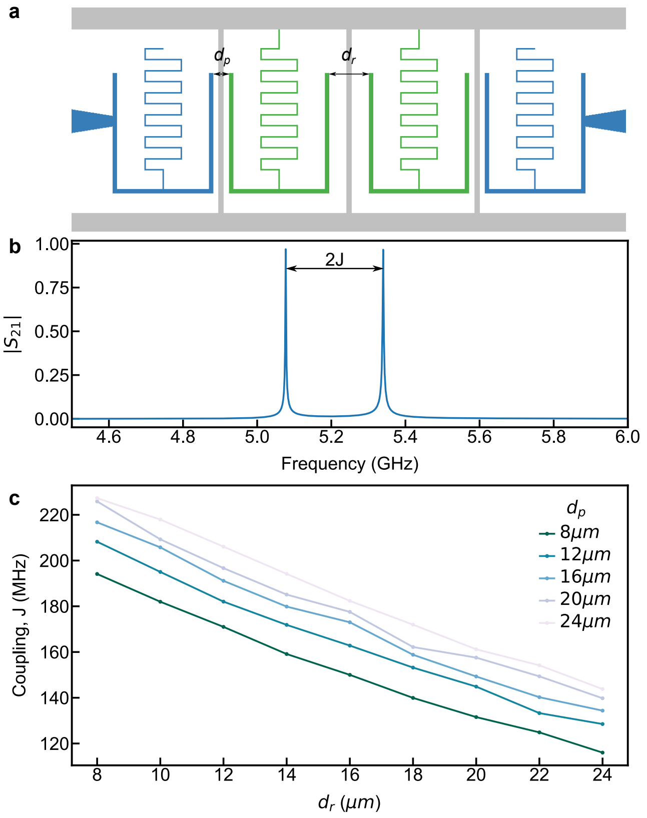

Supplementary Figure S6. **Coupling calibration for rectangular CCA geometry.** **a.** Schematic of the calibration design, with the cavities in green, the coupling ports in blue and the ground plane in grey. d_p is the distance between the ports (ghosts) and cavities, and d_r is the distance between the cavities. **b.** Simulation using Sonnet software of the transmission amplitude $|S_{21}|$ through the dimer as a function of the excitation frequency. The coupling, J, is extracted from the frequency splitting between the two modes frequencies. **c.** Coupling extracted from Sonnet simulations as a function of d_r for different values of d_p, assuming $L_{\mathrm{k},\square} = 100$ pH/$\square$.

port. $\hat{H}$ is the Hamiltonian of the system under study; in this case, we work with the normal Hamiltonian Eq. (B7) for the sake of simplicity. In the following, we assume that the internal dissipation rate, κ_{int}, is the same for all cavities.

One can write the previous equation in the steady state regime where $\hat{a}_n(t) = \hat{a}_n \exp(-i\omega t)$ and $\hat{a}_{\mathrm{in}}(t) = \hat{a}_{\mathrm{in}} \exp(-i\omega t)$, resulting in,

$$\begin{aligned} &\hat{a}_n \left(\Delta - j\frac{\kappa_n}{2}\right) + J\left(\hat{a}_{n+1}\left(1-\delta_{n,N}\right) + \hat{a}_{n-1}\left(1-\delta_{n,1}\right)\right) \\ &- j\sqrt{\kappa_{\mathrm{ext}}}\left(\delta_{n,1}\hat{a}_{\mathrm{in,L}} + \delta_{n,N}\hat{a}_{\mathrm{in,R}}\right) \\ &- j\sqrt{\kappa'_{\mathrm{ext}}}\left(\delta_{n,2}\hat{a}_{\mathrm{in,L}} + \delta_{n,N-1}\hat{a}_{\mathrm{in,R}}\right) = 0, \end{aligned} \tag{E2}$$

where $\Delta = \omega_r - \omega$ is the detuning between the probe frequency $\omega/2\pi$ and ω_r. We can now use the two input/output relations,

$$\hat{a}_{\mathrm{in,L(R)}} + \hat{a}_{\mathrm{out,L(R)}} = \sqrt{\kappa_{\mathrm{ext}}}\hat{a}_{1(N)} \tag{E3}$$

$$\hat{a}_{\mathrm{in,L(R)}} + \hat{a}_{\mathrm{out,L(R)}} = \sqrt{\kappa'_{\mathrm{ext}}}\hat{a}_{2(N-1)} \tag{E4}$$

where $\hat{a}_{\mathrm{out,L(R)}}$ is the output field on the left (right) side of the CCA. Note that the two input/output relations use the same input/output fields. By inserting Eqs. (E3) and (E4) into Eq. (E2) one obtains,

$$\begin{aligned} &\hat{a}_n \left(\Delta - j\frac{\kappa_n}{2}\right) + J\left(\hat{a}_{n+1}\left(1-\delta_{n,N}\right) + \hat{a}_{n-1}\left(1-\delta_{n,1}\right)\right) \\ &- j\sqrt{\kappa_{\mathrm{ext}}}\sqrt{\kappa'_{\mathrm{ext}}}\left(\delta_{n,1}\hat{a}_2 + \delta_{n,2}\hat{a}_1\right) \\ &- j\sqrt{\kappa_{\mathrm{ext}}}\sqrt{\kappa'_{\mathrm{ext}}}\left(\delta_{n,N-1}\hat{a}_N + \delta_{n,N}\hat{a}_{N-1}\right) \\ &- j\sqrt{\kappa_{\mathrm{ext}}}\sqrt{\kappa'_{\mathrm{ext}}}\left(\delta_{n,1}\hat{a}_{\mathrm{out,L}} + \delta_{n,N}\hat{a}_{\mathrm{out,R}}\right) \\ &- j\sqrt{\kappa_{\mathrm{ext}}}\sqrt{\kappa'_{\mathrm{ext}}}\left(\delta_{n,2}\hat{a}_{\mathrm{out,L}} + \delta_{n,N-1}\hat{a}_{\mathrm{out,R}}\right) = 0, \end{aligned} \tag{E5}$$

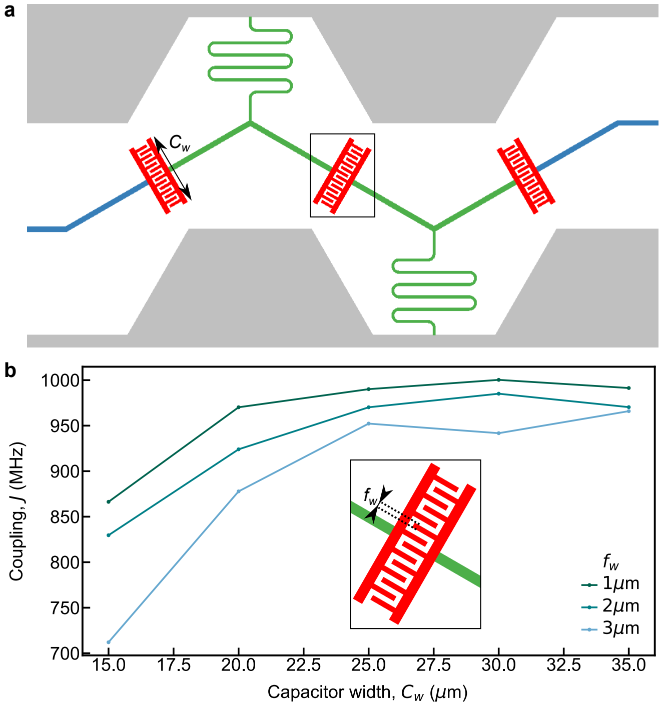

Supplementary Figure S7. **Coupling calibration for hexagonal CCA geometry. a.** Schematic of the calibration design, with the cavities in green, the coupling ports in blue, the capacitors that are swept through the simulation in red, and the ground plane in grey. C_w is the coupling capacitor width. **b.** Coupling extracted from Sonnet simulations as a function of C_w for different values of, f_w, representing the finger and gap width. The inset shows a zoom-in of the capacitor from panel **a**, assuming $L_{\mathrm{k},\square} = 100$ pH/$\square$.

This allows us to write down the non-Hermitian Hamiltonian in the field basis $A = \{\hat{a}_1, \hat{a}_2, \ldots, \hat{a}_n\}$,

$$\frac{\boldsymbol{H}_{\boldsymbol{n}}^{\text{Non-Herm}}}{\hbar} = \begin{pmatrix} \omega_r - j\frac{\kappa_1}{2} & J - j\sqrt{\kappa'_{\text{ext}}}\sqrt{\kappa_{\text{ext}}} & 0 & \cdots & \cdots & 0 \\ J - j\sqrt{\kappa'_{\text{ext}}}\sqrt{\kappa_{\text{ext}}} & \omega_r - j\frac{\kappa_2}{2} & J & \ddots & \ddots & \vdots \\ 0 & J & \omega_r - j\frac{\kappa_3}{2} & J & \ddots & \vdots \\ \vdots & \ddots & \ddots & \ddots & \ddots & \vdots \\ \vdots & \ddots & \ddots & \ddots & \omega_r - j\frac{\kappa_{N-1}}{2} & J - j\sqrt{\kappa'_{\text{ext}}}\sqrt{\kappa_{\text{ext}}} \\ 0 & \cdots & \cdots & \cdots \; J - j\sqrt{\kappa'_{\text{ext}}}\sqrt{\kappa_{\text{ext}}} & & \omega_r - j\frac{\kappa_N}{2} \end{pmatrix}. \tag{E6}$$

Despite this non-Hermitian has been derived for a uniform CCA, this procedure is valid for any CCAs discussed in the manuscript. We use this non-Hermitian Hamiltonian to extract the dissipation properties of the resonators in the CCA. Before moving on to this part, we show below how to obtain the scattering coefficients of any CCA. One can conveniently define the scattering coefficients as [5],

$$S_{kl} = \frac{a_l^{\text{out}}}{a_k^{\text{in}}} = j\sqrt{\kappa_{\text{ext,k}}\kappa_{\text{ext,l}}}\left[\boldsymbol{M}^{-1}\right]_{kl} - \delta_{kl}, \tag{E7}$$

where $\kappa_{\text{ext,j}}$ and $\kappa_{\text{ext,l}}$ are the dissipation rates to the output, k, and input, l, measurement ports, respectively. $[\boldsymbol{M}]$ is the equation of motion matrix defined as,

$$[\boldsymbol{M}] = [\boldsymbol{H}_{\boldsymbol{n}}^{\text{Non-Herm}}] - \omega\mathbb{1}. \tag{E8}$$

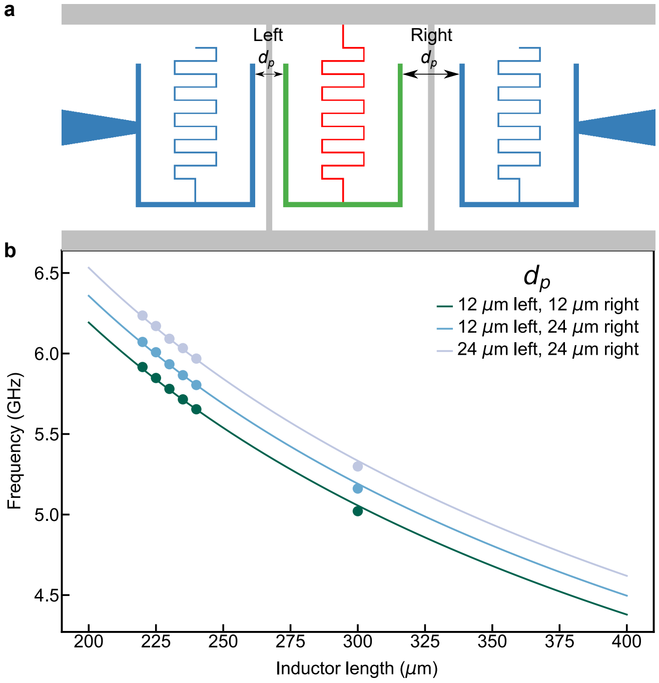

Supplementary Figure S8. **Calibration of the resonant frequency for multigap CCAs. a.** Schematic of the calibration design, with the capacitor of the cavity in green, the inductor swept through the simulation in red, the coupling ports in blue, and the ground plane in grey. **b.** Simulated resonant frequency of the cavity as a function of the length of the inductor for different spacing to coupling ports, d_p, using Sonnet software and assuming $L_{k,\square} = 100$ pH/□. The dots represent the resonant frequencies extracted from the simulation and the lines are fits with Eq. (20).

Given our choice of coupling ports, we only measure the scattering parameters S_{N1}, S_{11}, S_{NN} and S_{1N}, which we call with the standard notation, S_{21}, S_{11}, S_{22} and S_{12}.

2. Extracting dissipations

In order to extract the dissipation parameters of the system, we use both the non-Hermitian Hamiltonian Eq. (E6) and the scattering matrix Eq. (E7). Fitting the full scattering matrix is challenging, due to the number of fitting parameters and the long computational time of the full scattering matrix. We proceed instead by extracting the dissipations mode by mode, modeling each eigenmode as a single resonator with a certain external coupling ($\kappa_{\text{ext}}^{\text{Mode}}$) and internal loss rate ($\kappa_{\text{int}}^{\text{Mode}}$). The reflection scattering parameter of a single mode can be defined as

$$S_{11} = 1 - \frac{\kappa_{\text{ext}}^{\text{Mode}}}{i\Delta + (\frac{\kappa_{\text{ext}}^{\text{Mode}}}{2} + \frac{\kappa_{\text{int}}^{\text{Mode}\star}}{2})} = S_{22}, \tag{E9}$$

where $\kappa_{\text{int}}^{\text{Mode}\star}$ is the extracted internal dissipation from the fit, which also takes into account the dissipation to the other coupled microwave waveguide, at the other end of the CCA. Hence, we have $\kappa_{\text{int}} = \kappa_{\text{int}}^{\text{Mode}\star} - \kappa_{\text{ext}}^{\text{Mode}}$, where κ_{int} is the internal dissipation to the environments.

We can fit the total modes' dissipation, $\kappa_{\text{tot}}^{\text{Mode}} = \kappa_{\text{int}}^{\text{Mode}} + \kappa_{\text{ext}}^{\text{Mode}}$ with the complex part of the eigenvalues of the non-Hermitian Hamiltonian, Eq. (E6), from which we can extract the internal and external dissipations of the cavities in the CCA. In Figs. S10 (**c**, **f** and **i**), we fit the extracted dissipations for some representative devices in the normal ($J_1 = J_2$), topologically trivial ($J_1 > J_2$) and topologically non-trivial ($J_1 < J_2$) coupling configurations. From this fit, we can observe a clear asymmetry in the dissipations to the coupling port: the lower frequency modes (lower passband) are less coupled than the upper frequency modes (upper passband). We establish that this effect is caused by, κ'_{ext} the coupling between the microwave port and the second closest resonator to the microwave ports. Even though $\kappa'_{\text{ext}} \approx 10\,\text{kHz} \ll \kappa_{\text{ext}}$, it has a significant effect on the modes amplitudes. This effect is also qualitatively observable in all measured transmission spectra in Fig. 2, Fig. 3, Fig. S20 and Fig. S21.

Appendix F: SSH

SSH states represent a unique category of symmetry protected topological states that manifest in 1D systems characterized by alternating hopping amplitudes. They have been originally introduced in the realm of condensed matter physics to describe the electronic structure of polyacetylene chains, a 1D organic polymer [6]. For a careful derivation of the SSH model and discussion of its properties, we refer the reader to [7, 8].

1. SSH model and influence of second neighbor coupling

In this section, we numerically model the devices measured in Fig. 3. Specifically, we study the evolution of the energy spectra of the SSH CCA as a function of the next nearest neighbor coupling, J', and the number of resonators, N. The CCA in the SSH coupling configuration is described by the Hamiltonian Eq. (C8) with $J_1 < J_2$. With the CCA in the SSH configuration and $J' = 0$, we expect the formation of bulk bands separated by $2|J_2 - J_1|$ and the presence of in-gap modes at the center of the bandgap physically localized at the edges of the CCA.

We are now going to consider the $J' \neq 0$ term in Eq. (C8) and study its effects on the spectrum of the CCAs. In Eq. (C8) of the main text, we observe that the action of J' in the Hamiltonian is proportional to τ_0, for $J'/\bar{J} < 30\%$ it should not influence qualitatively the topological properties of the system [9]. We first study how $J' \neq 0$ modifies the SSH CCA spectrum (Fig. S12). As already observed in the topologically trvial case (see Fig. S4) we observe a compression of the low passband

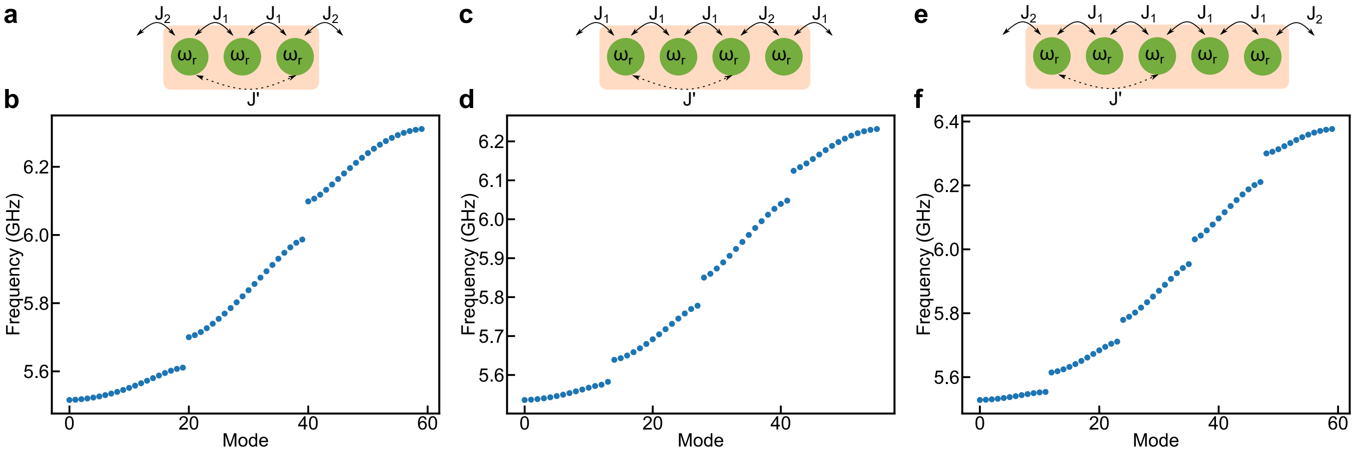

Supplementary Figure S9. **Mode dispersion for multigap CCAs. a.** (**c.**, **e.**) CCA schematic of the unit cell of multigap designs used in Fig. 2**e** with $M = 3$ (4, 5) cavities per unit cell, resulting in 2 (3, 4) bandgaps. **b.** (**d.**, **f.**) Simulated mode dispersion for the 2 (3, 4) bandgap devices with $N = 60$ (56, 60) cavities, simulated with the Hamiltonian Eq. (18) presented in Methods Sec. VI.

Supplementary Table S1. Table of quality factors of representative devices at low photon number. $\overline{\kappa_{\text{int}}^{\text{Mode}}}$ and $\overline{\kappa_{\text{ext}}^{\text{Mode}}}$ are the mean internal and external dissipation rates of the modes of a CCA. κ_{int} is the internal dissipation rate of the cavities in the CCA. κ_{ext} and κ'_{ext} are the dissipation rates to the coupling ports from the closest and second closest cavity to the microwave ports. ω_r is the frequency of the cavities in the CCA.

Figure/Device	$\omega_r/\overline{\kappa_{\text{int}}^{\text{Mode}}}$ ($\times 10^3$)	$\omega_r/\overline{\kappa_{\text{ext}}^{\text{Mode}}}$ ($\times 10^3$)	$\omega_r/\kappa_{\text{int}}$ ($\times 10^3$)	$\omega_r/\kappa_{\text{ext}}$ ($\times 10^3$)	$\omega_r/\kappa'_{\text{ext}}$ ($\times 10^3$)
Fig. 2**c**/Left	20	100	11	4	1242
Fig. 2**c**/Middle	70	137	10	2.32	75
Fig. 2**c**/Right	8.6	9.5	24.6	107	49
Fig. 2**d**/Left	62	63	22	1.67	122
Fig. 2**d**/Right	85	120	33	2.73	169
Fig. 2**e**/Top	37	116	26	1.27	67
Fig. 2**e**/Bottom	41	144	26	1.5	35
Fig. 3**d**/16	34	29	12	1.48	145
Fig. 3**f**/16	75	30	35	1.1	363

together with an expension of the upper passband as a function of J'. In addition, while increasing J' the SSH modes are also shifted with respect to the resonant frequency of the CCA resonators, $\omega_r/2\pi$ (see red line in Fig. S12), up to the point where they cannot distinguished from the bulk modes of the upper passband. J' also impacts the modes' spatial distribution, allowing for some photonic population to extends into the neighboring sub-cell, thereby breaking chiral symmetry, even in the absence of a τ_z term in the Hamiltonian in Eq. (C8). This effect becomes stronger as J' is increased (Fig. S12).

2. Amplitude of the SSH modes

In Figs. 3**d** and **f** one striking observation is represented by the drop in amplitude of the SSH modes for CCAs measured in transmission (S_{21}) as a function of the number of resonators, N. Here, we model this effect and show how it scales using the input/output formalism introduced in App. E (Eq. (E7)). In Fig. S13, we report the simulated $|S_{21}|$ in an SSH CCA characterized by $J_2/J_1 = 1.57$ as a function of the number of cavities, N. We clearly observe that, the amplitude of the SSH modes drops drastically as a function of N until the SSH modes are no more visible in transmission. This effect can be intuitively explained from the nature of the SSH modes. Indeed, due to their exponential localization at the edges of the CCA, their interaction occurs primarily through the overlap of the tails of their wavefunctions within the bulk region. As the bulk size increases, the overlap between these modes diminishes, leading to a reduction in hybridization between them. Consequently, the effective photon hopping rate at the frequency of the

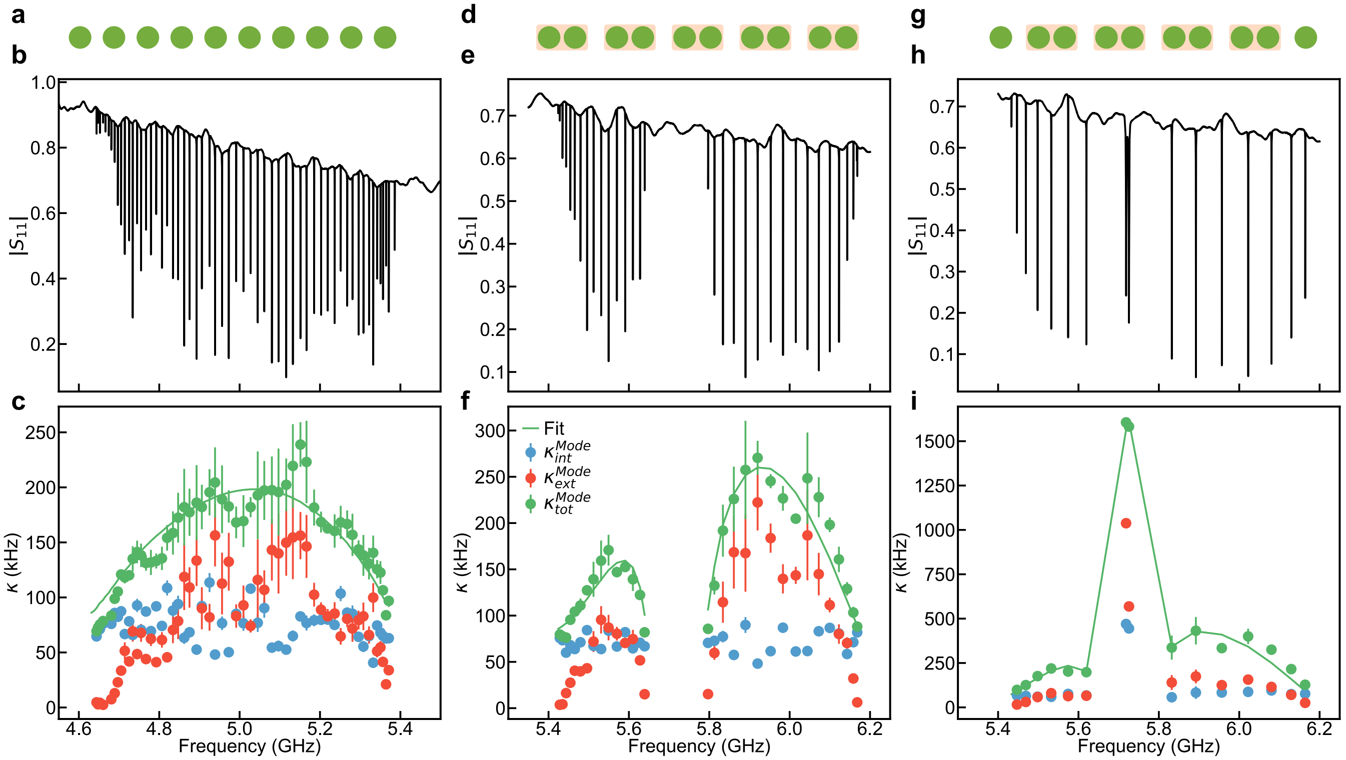

Supplementary Figure S10. **Fit of the dissipation for representative CCAs in the normal, trivial and topological configuration. a.** (**d.**, **g.**) CCA schematic in the normal (trivial, topological) configuration. **b.** (**e.**, **h.**) Magnitude of the reflection signal, $|S_{11}|$, of a CCA in the normal (trivial, topological) configuration with $N = 64$ (32,16), measured at low power. **c.** (**f.**, **i.**) Internal (blue), external (red) and total (green) dissipation rates fitted for each mode according to Eq. (E9). The green line is a fit of the total dissipation of the modes, $\kappa_{\rm tot}^{\rm Mode}$, according to the complex part of the eigenvalues of the non-Hermitian Hamiltonian Eq. (E6).

SSH modes, originating from the coupling of the two microwave ports on the side of the CCA, decreases, resulting in lower transmission. We anticipate a more rapid decrease in transmission with respect to the number of resonators, denoted as N, in the strongly localized configuration.

Appendix G: Disorder

1. Influence of resonator frequency scattering on the bulk modes

A qualitative way to understand how disorder affects the spectrum of the CCA can be implemented by simulating the CCA transmission, $|S_{21}|$, while introducing scattering on the resonant frequencies induced by Gaussian noise on the inductances, σ_L. In Fig. S14, we plot a simulation of the transmission amplitude, $|S_{21}|$, in a uniform CCA with $N = 50$. The CCA parameters are the one extracted from the CCA in Fig. 2**b** for different values of Gaussian noise σ_L. In Fig. S14 we refer to the applied noise as, $\sigma_{L\to z}$, the disorder induced by σ_L on the resonant frequency of the cavities in the CCA. When no disorder is applied, the amplitude of the modes follows the same trend as the eigenvalues of the complex part of the non-Hermitian HamiltonianEq. (E6). As we increase the disorder, we observe the appearance of ripples in the mode's amplitude and frequency deviation from what is reported in Fig. S14**a**. This is due to the fact that the resonators are not degenerate anymore. From this simulated trend of the transmission spectrum, we can safely establish that the disorder in our devices is below $\sigma_{L\to z}/f_r = 0.4\%$.

2. Influence of disorder on the SSH modes

Since the SSH modes are protected by chiral symmetry, they are very sensitive to the chiral symmetry breaking terms in Hamiltonian Eq. (3) of the main text. As discussed in the main text, terms proportional to τ_z will break topological protection. Such terms simply appear in the Hamiltonian due to frequency scattering between the resonators, which naturally arise due to fabrication imperfection. In Fig. S15, we simulate the effect of disorder on the SSH modes' hybridization, $\Delta_{\rm Topo}$, as a function of the number of resonators, N. The simulation is performed using the Hamiltonian Eq. (18) in Methods,

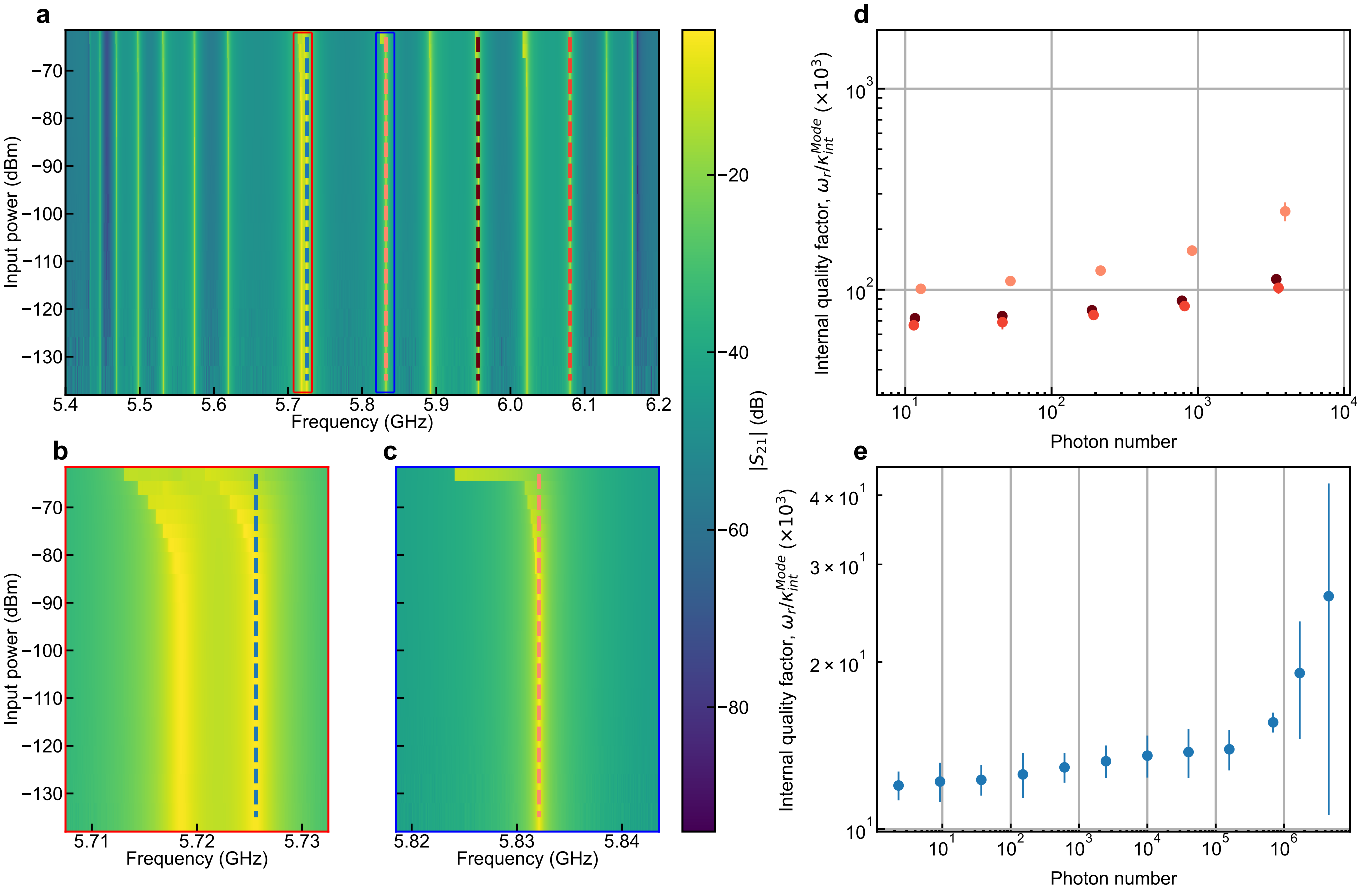

Supplementary Figure S11. **Power dependence of the CCAs.** **a.** Power scan of a CCA in the topological configuration with $J_2/J_1 = 1.57$ and $N = 16$ measured in transmission. **b.** Zoom-in of the power scan on the SSH modes. **c.** Zoom-in of the power scan on a mode of the bulk. The frequency span for both cuts is the same. **d.** Internal quality factors, $\omega_r/\kappa_{\rm int}^{\rm Mode}$, of representative bulk modes as a function of the estimated photon number in the modes. The fitted modes are highlighted by the three red dashed lines in panels **a** and **c**. **e.** Internal quality factors, $\omega_r/\kappa_{\rm int}^{\rm Mode}$, of a representative SSH mode as a function of the estimated photon number. The mode is highlighted by the blue dashed lines in panels **a** and **b**.

using the parameters of the device in the SSH configuration with $J_2/J_1 = 1.22$. The σ_L noise applied to the inductors induces both τ_z and τ_x type of disorder, impacting respectively the resonant frequency and the coupling of the resonators in the CCA. Although τ_z-type disorder breaks chiral symmetry, we anticipate that the SSH modes will still exhibit some degree of resilience against τ_x-type disorder.

We study three cases: 1) $\sigma_z \neq 0$ and $\sigma_x = 0$ (Fig. S15**a**), 2) $\sigma_z = 0$ and $\sigma_x \neq 0$ (Fig. S15**b**), and 3) $\sigma_z \neq 0$ and $\sigma_x \neq 0$ (Fig. S15**c**).

In Fig. S15**a**, we report the median of $\Delta_{\rm Topo}$ for different values of σ_z. For $\sigma_z = 0$, the disorder-free case, one expects $\Delta_{\rm Topo}$ to decay exponentially as a function N following Eq. (4). By introducing and increasing σ_z disorder, we observe a saturation of $\Delta_{\rm Topo}$ as a function of the number of cavities. This saturation value of $\Delta_{\rm Topo}$ increases non-uniformly as a function of disorder σ_z.

In Fig. S15**b**, we implement the same study as above but for τ_x type disorder. We can observe a considerable deviation from the expected noiseless case just for high enough values of σ_x and N.

In Fig. S15**c**, we apply both types of disorder at the same time. We keep the disorder σ_z constant but sweep σ_x. As expected, we observe a saturation of $\Delta_{\rm Topo}^N$ as a function of N, which is independent from the magnitude of σ_x.

In Fig. S16 we implement a similar study as in Fig. S15, with disorder on the next-nearest neighbor coupling, J', namely, σ'. With J' being proportional to τ_0 (Eq. (3) of the main text) σ'-disorder will result in σ_z-type disorder. In Fig. S16**a**, as in Fig. S15**b**, we observe a considerable deviation just for high enough values of σ' and N. In Fig. S16**b**, we apply the same disorders as in Fig. S16**a** but with constant resonant frequency disorder $\sigma_z/f_r = 0.2\%$, as in Fig. S15**c**. Again, here one can see that resonant frequency disorder, σ_z, dominates over the next-nearest neighbor coupling disorder, σ'.

This justifies why in the main text, we focus only on τ_z type disorder. The disorder on the inductance affects not

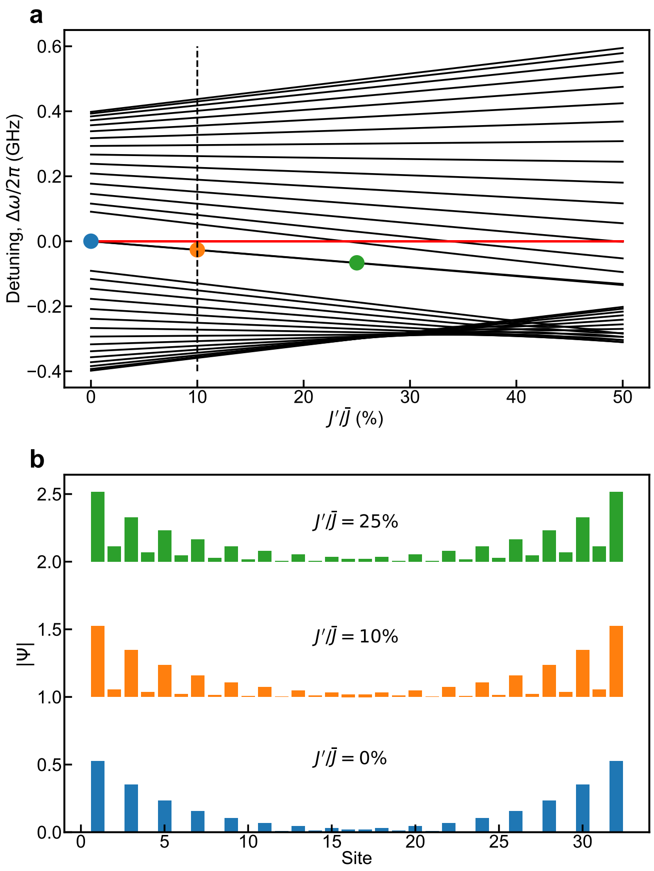

Supplementary Figure S12. **Influence of second neighbor coupling on the SSH modes. a.** Eigenvalues for a CCA with $N = 32$ and $M = 2$ in the topologically non-trivial coupling configuration, calculated according to Eq. (C7) as a function of $J'/\overline{J}$, where $\overline{J} = 1/2(J_1 + J_2)$. The modes frequencies are represented as a function of the detuning $\Delta\omega = \omega - \omega_r$. The red line represents $\omega = \omega_r$. For this simulation we have used, $J_1/2\pi = 160\,\text{MHz}$, $J_2/2\pi = 240\,\text{MHz}$ and $\omega_r/2\pi = 5\,\text{GHz}$. The vertical dashed line indicates the typical $J'/\overline{J}$ for the rectangular CCA geometry. **b.** Simulation results depicting the norm of the spatial distribution of the symmetric SSH mode for various strengths of second neighbor coupling are presented in **a**.

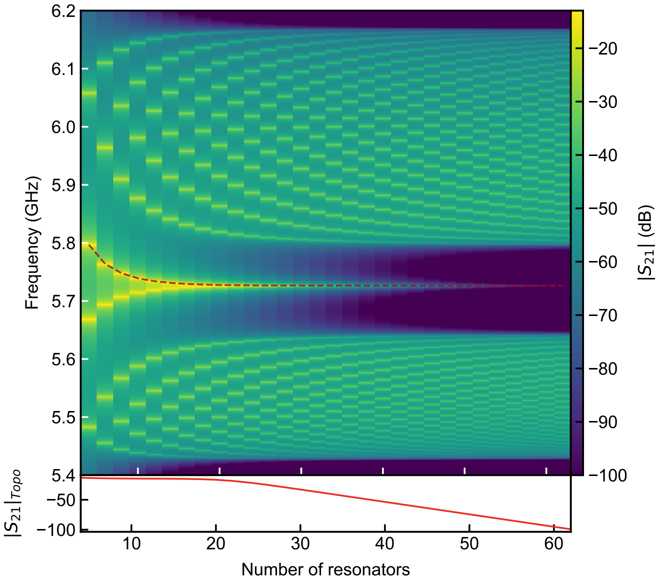

Supplementary Figure S13. **Evolution of the CCA transmission in the SSH configuration as a function of the number of cavities.** Input/output simulation using Eq. ((E7)) with Hamiltonian Eq. (18) of the evolution of the amplitude of the CCA transmission, $|S_{21}|$, in a CCA with $J_2/J_1 = 1.57$ as a function of the numbers of resonators, N. The inset with the red line shows the evolution of the transmission of the symmetric SSH mode as a function of the number of resonators, N.

only the SSH modes splitting but also the spatial profile of the SSH modes as can be observed in Fig. S17. Here, the effect of σ_L disorder is studied by employing Hamiltonian Eq. (18) for $N = 16$ cavities with $J_2/J_1 = 1.57$. In a disorder-free case, we expect the SSH modes to hybridize and form a symmetric and an antisymmetric superposition, presenting equal norm on the different CCA site ($|\Psi_S| = |\Psi_{AS}|$, see Fig. S17**a**). This symmetry is also observed in the simulation according to Eq. (E7) introduced in App. E of the phase of the reflected signal at the microwave ports, $\text{Arg}(S_{11}, S_{22})$ (see Fig. S17**b**). From both ports, we find equal phase shifts for the symmetric and antisymmetric SSH modes.

In the remaining panels of Fig. S17, we report two independent realization of disordered scenarios of the SSH modes with $\sigma_{L\to z} = 10\,\text{MHz}$. In Fig. S17**c** and **e**, we show $|\Psi_{AS}|$ and ($|\Psi_S|$) for the two instances of disorder, respectively. We observe that the disorder will randomly change the localization of the modes, making them asymmetric. This behavior is again reflected in $\text{Arg}(S_{11}, S_{22})$, as reported in Fig. S17**d** and Fig. S17**f**, respectively corresponding to SSH mode profile in Figs. S17**c** and **e**. We can observe a clear asymmetry in the phase shifts from the two microwave ports, which suggest a stronger localization of both SSH modes, on one side or the other of the CCA, as opposed to the disorder-free case (Fig. S17**b**).

3. Influence on the parameter estimation

The estimation of the parameters extracted from the spectra of the CCAs using the model described in Methods Sec. VI can be affected by disorder. In order to get an estimation of the error on the extracted parameters, we perform a statistical analysis of the fitting method implemented as explained here below (Fig. S18).

We simulate using the Hamiltonian Eq. (18) of a CCA with $N = 32$ cavities for the two SSH coupling configurations introduced in the main text ($J_2/J_1 = 1.22$ and $J_2/J_1 = 1.57$). We introduce a frequency scattering on the cavities by applying a Gaussian noise, σ_L, on the inductances of the resonators. For each value of disorder σ_L, we perform 500 fits. In Fig. S18, we report the fitted

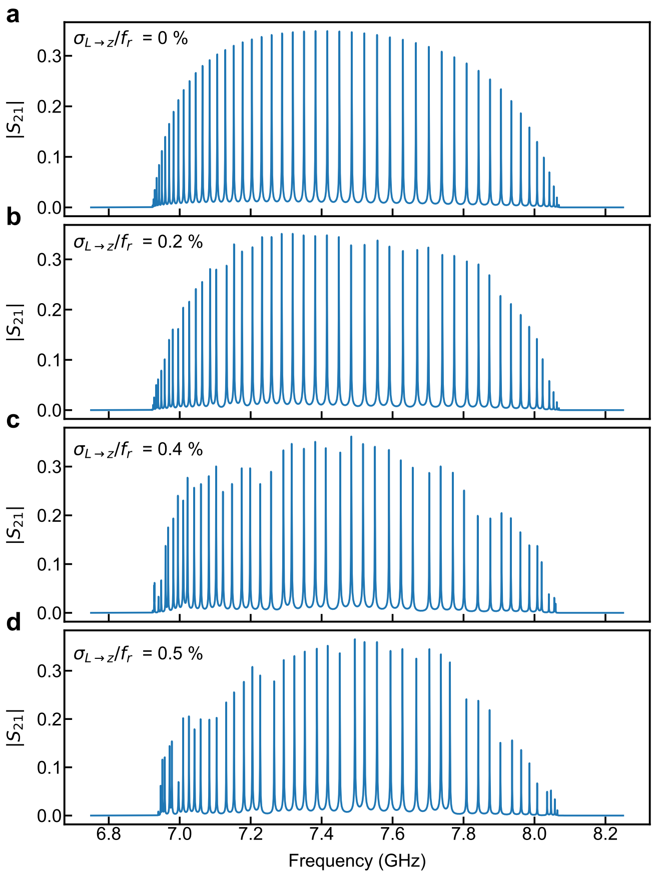

Supplementary Figure S14. **Simulation of the transmission, $|S_{21}|$, in a CCA with Gaussian disorder applied to the inductances.** From **a.** to **d.**, transmission through CCAs with $N = 50$ resonators ($M = 1$) with disorder values of $\sigma_{L\to z}/f_r = 0\%$ (**a**), $\sigma_{L\to z}/f_r = 0.2\%$ (**b**), $\sigma_{L\to z}/f_r = 0.4\%$ (**c**) and $\sigma_{L\to z}/f_r = 0.5\%$ (**d**), applied to the inductances of the resonators. The simulation is performed using Hamiltonian Eq. (18) using the parameters of the device presented in Fig. 3**b** and in Tab. S2.

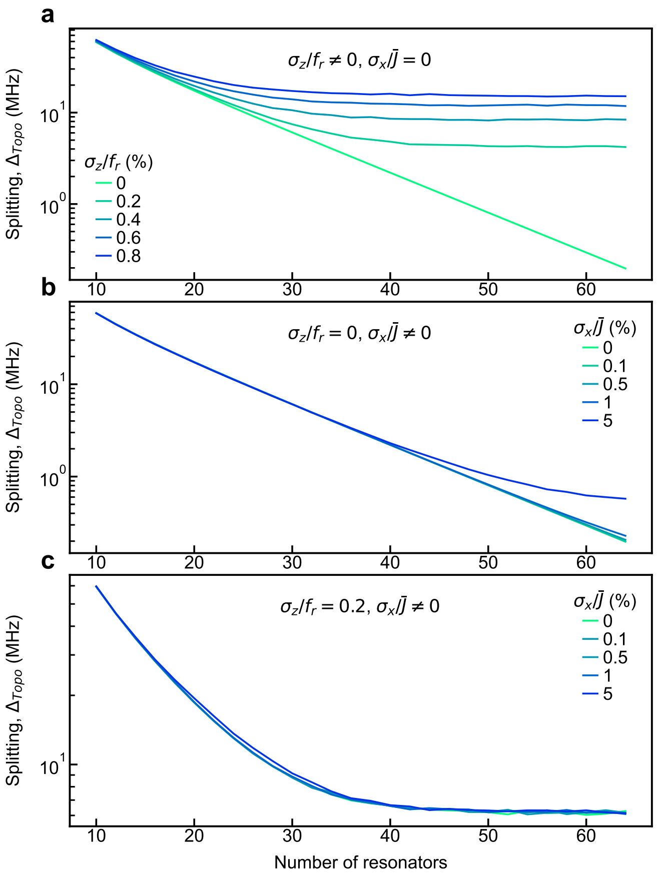

Supplementary Figure S15. **Simulation of the influence of σ_z and σ_x disorder on the SSH modes splittings, $\Delta_{\mathbf{Topo}}$.** The simulations are performed with a Hamiltonian on a CCA in the SSH configuration with $J_2/J_1 = 1.22$. **a.** Splittings of the SSH modes, Δ_{Topo}, as a function of the number of resonators, N, for different values of σ_z disorder ($\sigma_x = 0$). **b.** Δ_{Topo} as a function of N for different values of σ_x ($\sigma_z = 0$). **c.** Δ_{Topo} as a function of N for different values of σ_x disorder ($\sigma_z = 0.2\% f_r$).

parameters as a function of σ_L. For all parameters except the inductance, which is a fixed-fitting parameter, we observe a deviation from the initial parameters of the simulated CCAs in absence of disorder. For the typical disorder extracted in our study $\sigma_L/L_g = 0.44^{+0.09}_{-0.06}$ (highlighted by the black dashed lines in Figs. S18**b**, **c** and **d**), we find an error of approximately 10 aF for the capacitances and 0.1% for the capacitance ratios (Figs. S18**e** and **f**).

Appendix H: Time-domain measurements

In this section, we describe how the time-resolved measurements of the SSH modes are performed and analyzed.

As described in the main text, the measurement is implemented by sending a Gaussian pulse from one of the edges of the CCA at a frequency in the middle of the two SSH modes. The signal is acquired throughout the full pulse sequence, i.e. before and after the excitation pulse, from both sides of the CCA. It is then demodulated at the frequency at which the pulse is sent. The demodulated reflected or transmitted signal, away from the pulse, is expected to take the form [10]

$$|S_{11}|(t) = e^{-\kappa_1 t}|\cos(gt + \varphi_1)|, \tag{H1}$$

$$|S_{21}|(t) = e^{-\kappa_2 t}|\sin(gt + \varphi_2)|, \tag{H2}$$

with κ_1 and κ_2 being the total dissipations of SSH modes 1 and 2. We use this equation to fit the beating profiles presented in Fig. 4**c** of the main text.

The pulse width and shape need to be carefully calibrated to avoid spurious frequency components exciting the other modes of the CCA. In Fig. S19, we show two cases of a Gaussian pulse applied to a CCA with $N = 26$ and $J_2/J_1 = 1.22$ (shown in Fig. 4 of the main text) for different pulse length of 24 ns(Fig. S19**b**) and

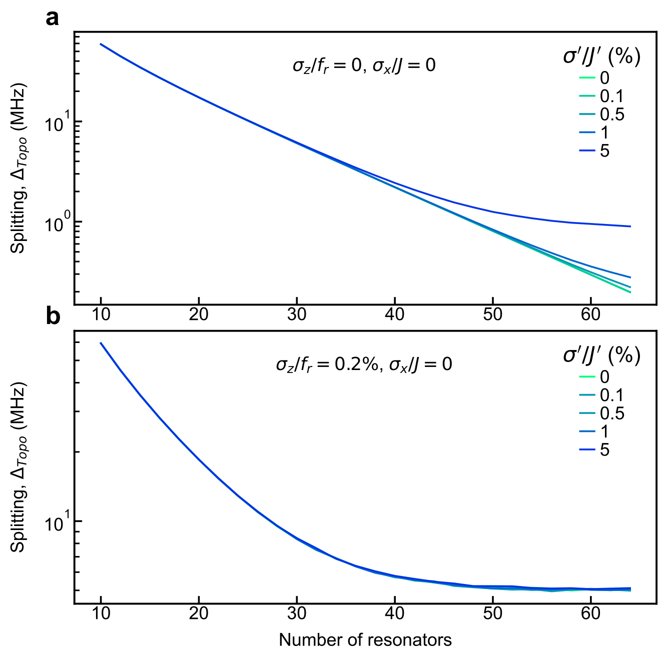

Supplementary Figure S16. **Simulation of the influence of next-nearest neighbor coupling disorder, σ', on the SSH modes splittings, Δ_{Topo}.** The simulations are performed with a Hamiltonian on a CCA in the SSH configuration with $J_2/J_1 = 1.22$. **a.** Splittings of the SSH modes, Δ_{Topo}, as a function of the number of resonators, N, for different values of σ' disorder ($\sigma_z = 0$ and $\sigma_x = 0$). **c.** Δ_{Topo} as a function of N for different values of σ' disorder ($\sigma_z = 0.2\% f_r$).

144 ns (Fig. S19**c**) as a function of the pulse frequency. The beating pattern developed by the shorter pulse is larger because its frequency components can excite the SSH modes even when detuned from the midpoint of the two SSH modes. For the pulse with longer length, the beating pattern is only happening, as expected, between the SSH modes.

Appendix I: Extra data

In this section, we show measurements of some extra devices similar to the one shown in the main text.

In Fig. S20, we show devices associated with Fig. 2**c**. In Fig. S20**a**, we display spectra of devices with $M = 1$ and $N = 16, 32$ and 64 cavities with the rectangular design.

Figs. S20**b** and **c**, exhibit several measurements of CCAs featuring hexagonal geometry with $M = 1$ and $N = 26, 42$ and 64. In panel **b**, we can observe multiple spurious modes starting from 11 GHz on three distinct CCAs. In panel **c**, we report the amplitude of the transmission, $|S_{21}|$ of the same device measured during a separate cooldown. Here, while the additional modes at 11 GHz are no longer visible, other spurious effects emerge around 9.5 GHz. These observations lead us to conclude that these spurious modes are not intrinsic to the devices themselves.

In Fig. S21, we show $|S_{21}|$ for all the SSH-CCA used in the disorder analysis reported in Fig. 4.

Appendix J: Measurement setup

A schematic of the measurement setup is depicted in Fig. S22. A simplified version of the cryogenic setup is presented in Fig. S22**a**. The device is thermally anchored to the mixing chamber plate of a commercial dry dilution cryostat (Bluefors-LD) at a temperature of 10 mK. Multiple devices can be measured in a single cooldown both in reflection and transmission making use of cryogenic circulators (LNF-CICI48A) and cryogenic switches (Radiall R577432000). The input lines are attenuated with cryogenic attenuators at different stages of the cryostat, as reported in Fig. S22**a**. The signal output from the device goes through one circulator and three isolators before being amplified at 4K with a HEMT amplifier (LNF-LNC4-8C). The signal is then further amplified at room temperature with a low-noise amplifier (Agile AMT-A0284). The full scattering matrix of the devices is characterized using an R&S ZNB20 vector network analyzer (VNA) (Fig. S22**b**).
We used arbitrary waveform generators (AWG) and Digitizers from an OPX+ from Quantum Machine, for implementing time-resolved measurements. This measurement consists of sending a Gaussian pulse at an intermediate frequency from the AWG OPX+ (see Fig. S22**c**); it is then up-converted at room temperature with an IQ mixer in the Octave (Quantum-machine) and sent down to the sample. After amplification, the reflected and transmitted signals are down-converted by an IQ mixer in the Octave (Quantum-machine) and then digitized in the OPX+ module. Finally, the signal is demodulated to DC in slices of 4 ns and averaged over 20,000 repetitions.

Appendix K: Tables of parameters

[1] U. Vool and M. Devoret, Introduction to quantum electromagnetic circuits, International Journal of Circuit Theory and Applications **45**, 897–934 (2017).
[2] M. Mirhosseini, E. Kim, V. S. Ferreira, M. Kalaee, A. Sipahigil, A. J. Keller, and O. Painter, Superconducting metamaterials for waveguide quantum electrodynamics, Nature Communications **9** (2018).
[3] Y. Liu and A. A. Houck, Quantum electrodynamics near a photonic bandgap, Nature Physics **13**, 48 (2017).

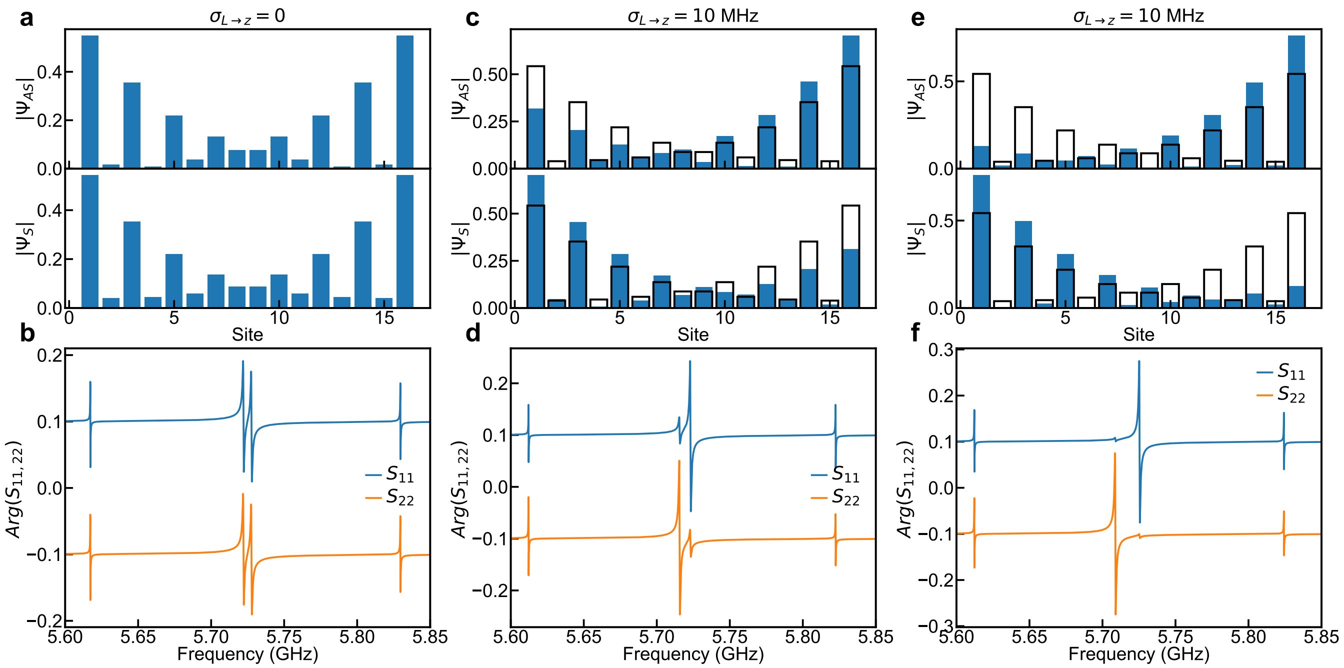

Supplementary Figure S17. **Simulation of the influence of disorder on the SSH modes localization. a.** (**c.**, **e.**) Simulated mode distribution of the norm of the antisymmetric ($|\Psi_{AS}|$) and symmetric ($|\Psi_S|$) SSH modes, for different disorder realizations. The black boxes in **c** and **e** represent the mode distribution in the disorder-free case **a**. **b.** (**d.**,**f.**) Simulation of $\text{Arg}(S_{11})$ and $\text{Arg}(S_{22})$ reflection spectrum for the same disorder realization as in **a** (**c**, **e**). The simulations are performed on CCAs with $N = 16$ in the same configuration as the device presented in Fig. 3**f** of the main text.

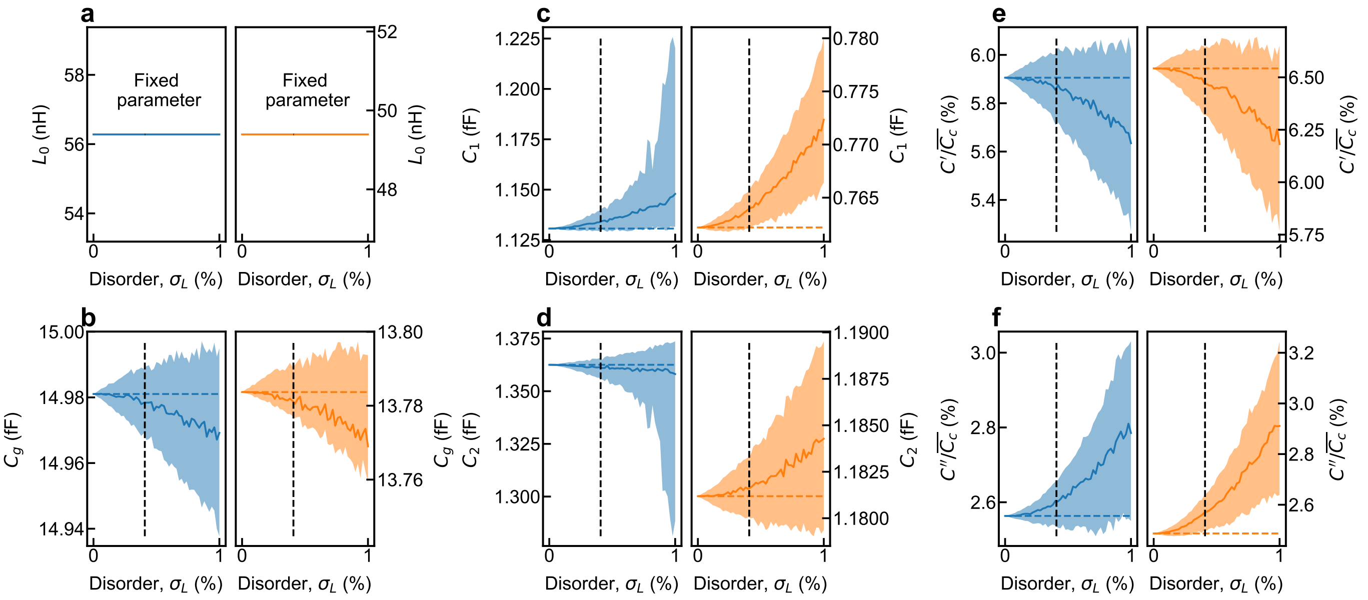

Supplementary Figure S18. **Simulation of the influence of disorder on the estimation of parameters.** Statistical simulation of the influence of disorder on the inductance σ_L, for a $N = 32$ CCA, on the estimation of **a.**, the inductance to ground, L_g, **b.**, the capacitance to ground C_g, **c.**, the intracell capacitance C_1, **d.**, the intercell capacitance C_2, **e.**, the second nearest neighbor coupling capacitance ratio $C'/\overline{C_c}$ and **f.**, the third nearest neighbor coupling capacitance ratio $C''/\overline{C_c}$. The left (blue) and right (orange) panels of each subfigure represent the weak and strong coupling configuration $J_2/J_1 = 1.22$ and $J_2/J_1 = 1.57$, respectively, as in Fig. 3. The continuous lines represent the median of the extracted parameters, and the shaded areas represent the 1σ uncertainty. The horizontal dashed lines represent the initial parameters set in the simulation.

Supplementary Table S2. Table of extracted CCA parameters for Fig. 2. The parameters are defined as follow: N is the number of cavities in the CCA. $\vec{L_g}$ is the inductance or inductances to ground if $M > 2$. $\vec{C_g}$ is the capacitance to ground or capacitances to ground if $M > 2$. $\vec{C_c}$ is the coupling or coupling capacitances if $M > 1$. $\omega_r/2\pi$ is the resonant frequency of the cavities. Z_r is the estimated impedance of the cavities in the array. $\vec{J}$ is the coupling or couplings between the cavities if $M > 1$. $C'/\bar{C}_c$ is the second nearest neighbor capacitance over $\overline{C_c}$. $C''/\bar{C}_c$ is the third nearest neighbor capacitance over $\overline{C_c}$.

Panel	N	$\vec{L_g}$ (nH)	$\vec{C_g}$ (fF)	$\vec{C_c}$ (fF)	$\omega_r/2\pi$ (GHz)	Z_r (kΩ)	$\vec{J}$ (MHz)	$C'/\bar{C}$ (%)	$C''/\bar{C}$ (%)
**b**	25	30.25	13.02	1.14	7.43	1.52	280	5.98	1.24
	50	30.25	13.02	1.14	7.43	1.52	280	5.91	1.16
	100	30.25	13	1.14	7.43	1.52	280	5.88	1.23
**c**	51	65.8	16.09	0.4	4.77	2.02	57	6.73	2.03
	64	65.8	12	0.8	5.32	2.34	164	4.9	1.89
	64	18.8	10.9	6.09	8.09	0.9	1164	0.01	0.01
**d**	32	56.56	16.36	1.48<br>1.24	4.86	1.72	191<br>160	5.91	2.03
	32	48.104	14.02	1.20<br>0.77	5.75	1.73	217<br>141	6.24	2.57
**e**	60	46.43<br>44.05	13.93<br>14.03	1.19<br>1.19<br>0.76	5.874	1.7	214<br>226<br>144	5.99	1.33
	56	46.43<br>48.88	13.63<br>13.78	1.03<br>1.03<br>0.86<br>1.03	5.91	1.77	197<br>168<br>164<br>160	5.31	0.76
	60	46.43<br>44.05	13.36<br>14.83	1.16<br>1.16<br>1.16<br>1.16<br>0.81	5.91	1.77	200<br>192<br>192<br>190<br>164	6.02	0.68

Supplementary Table S3. Table of extracted CCA parameters for Fig. 3. The parameters are defined as follow: N is the number of cavities in the CCA. $\vec{L_g}$ is the inductance or inductances to ground if $M > 2$. $\vec{C_g}$ is the capacitance to ground or capacitances to ground if $M > 2$. $\vec{C_c}$ is the coupling or coupling capacitances if $M > 1$. $\omega_r/2\pi$ is the resonant frequency of the cavities. Z_r is the estimated impedance of the cavities in the array. $\vec{J}$ is the coupling or couplings between the cavities if $M > 1$. $C'/\bar{C}_c$ is the second nearest neighbor capacitance over $\overline{C_c}$. $C''/\bar{C}_c$ is the third nearest neighbor capacitance over $\overline{C_c}$.

Panel/Batch	N	$\vec{L_g}$ (nH)	$\vec{C_g}$ (fF)	$\vec{C_c}$ (fF)	$\omega_r/2\pi$ (GHz)	Z_r (kΩ)	$\vec{J}$ (MHz)	$C'/\bar{C}$ (%)	$C''/\bar{C}$ (%)
**d**/A	16	56.28	14.94	1.13<br>1.35	5.1	1.8	168<br>200	6.04	2.32
	32	56.28	14.99	1.13<br>1.37	5.09	1.79	166<br>201	5.78	2.88
	64	56.28	15	1.13<br>1.36	5.09	1.79	166<br>200	5.89	2.47
**e**/B	16	49.39	13.64	0.75<br>1.17	5.75	1.78	141<br>218	6.83	2.42
	32	49.39	13.89	0.76<br>1.19	5.70	1.76	140<br>215	6.34	2.55
	64	49.39	13.81	0.76<br>1.18	5.72	1.77	141<br>216	6.44	2.47

[4] D. Walls and G. J. Milburn, eds., *Quantum Optics* (Springer, Berlin, Heidelberg, 2008).

[5] O. Naaman and J. Aumentado, Synthesis of Parametrically Coupled Networks, PRX Quantum **3**, 020201 (2022).

[6] W. P. Su, J. R. Schrieffer, and A. J. Heeger, Soliton excitations in polyacetylene, Physical Review B **22**, 2099 (1980).

[7] J. Dalibard, *College de France Lecture 2018: Topological Matter Explored with Quantum Gases*, Collège De France

Supplementary Table S4. Table of extracted CCA parameters for Fig. 4. The parameters are defined as follow: N is the number of cavities in the CCA. $\vec{L_g}$ is the inductance or inductances to ground if $M > 2$. $\vec{C_g}$ is the capacitance to ground or capacitances to ground if $M > 2$. $\vec{C_c}$ is the coupling or coupling capacitances if $M > 1$. $\omega_r/2\pi$ is the resonant frequency of the cavities. Z_r is the estimated impedance of the cavities in the array. $\vec{J}$ is the coupling or couplings between the cavities if $M > 1$. $C'/\bar{C}_c$ is the second nearest neighbor capacitance over $\overline{C_c}$. $C''/\bar{C}_c$ is the third nearest neighbor capacitance over $\overline{C_c}$.

Batch	N	$\vec{L_g}$ (nH)	$\vec{C_g}$ (fF)	$\vec{C_c}$ (fF)	$\omega_r/2\pi$ (GHz)	Z_r (kΩ)	$\vec{J}$ (MHz)	$C'/\bar{C}$ (%)	$C''/\bar{C}$ (%)
	14	56.22	14.92	1.18<br>1.41	5.09	1.79	174<br>207	5.17	0.77
	18	56.22	14.82	1.16<br>1.41	5.11	1.79	173<br>209	4.72	0.9
C	22	56.22	14.67	1.21<br>1.33	5.13	1.81	182<br>200	5.78	0.5
	26	56.22	15.06	1.18<br>1.44	5.06	1.78	171<br>208	5.51	1.24
	30	56.22	14.95	1.17<br>1.42	5.09	1.79	172<br>208	5.29	0.74
	14	56.05	13.98	0.78<br>1.20	5.33	1.87	131<br>202	5.69	0.44
	18	56.05	13.81	0.9<br>1.07	5.36	1.88	155<br>184	6.29	0
D	22	56.05	13.77	0.76<br>1.18	5.37	1.88	133<br>204	5.6	0.4
	26	56.05	14.01	0.78<br>1.21	5.33	1.87	132<br>203	6.13	1.16
	30	56.05	13.95	0.77<br>1.20	5.34	1.87	132<br>203	5.75	0.72
	32	48.10	13.84	0.76<br>1.18	6.07	1.66	150<br>230	6.33	2.43
	32	48.10	13.78	0.76<br>1.18	6.08	1.67	150<br>230	6.35	2.41
E	32	48.10	13.65	0.75<br>1.17	6.11	1.67	150<br>231	6.41	2.37
	32	48.10	13.78	0.76<br>1.18	6.07	1.66	150<br>230	6.36	2.41
	32	48.10	13.87	0.76<br>1.19	6.08	1.66	150<br>230	6.25	2.43
	32	48.10	13.87	0.76<br>1.18	6.1	1.67	151<br>232	6.58	2.28
	32	48.10	13.84	0.76<br>1.18	6.31	1.60	155<br>238	6.38	2.52
	32	48.10	13.79	0.76<br>1.18	6.32	1.61	155<br>239	6.33	2.51
F	32	48.10	13.68	0.76<br>1.17	6.35	1.61	156<br>240	6.33	2.54
	32	48.10	13.84	0.77<br>1.19	6.32	1.61	155<br>239	6.32	2.52
	32	48.10	13.83	0.77<br>1.19	6.30	1.60	155<br>238	6.19	2.56
	32	48.10	13.72	0.76<br>1.18	6.30	1.60	155<br>237	6.26	2.72

(2018).

[8] J. K. Asbóth, L. Oroszlány, and A. Pályi, *A Short Course on Topological Insulators*, Lecture Notes in Physics, Vol. 919 (Springer International Publishing, Cham, 2016).

[9] B. Pérez-González, M. Bello, Á. Gómez-León, and G. Platero, Interplay between long-range hopping and disorder in topological systems, Physical Review B **99**, 035146 (2019).

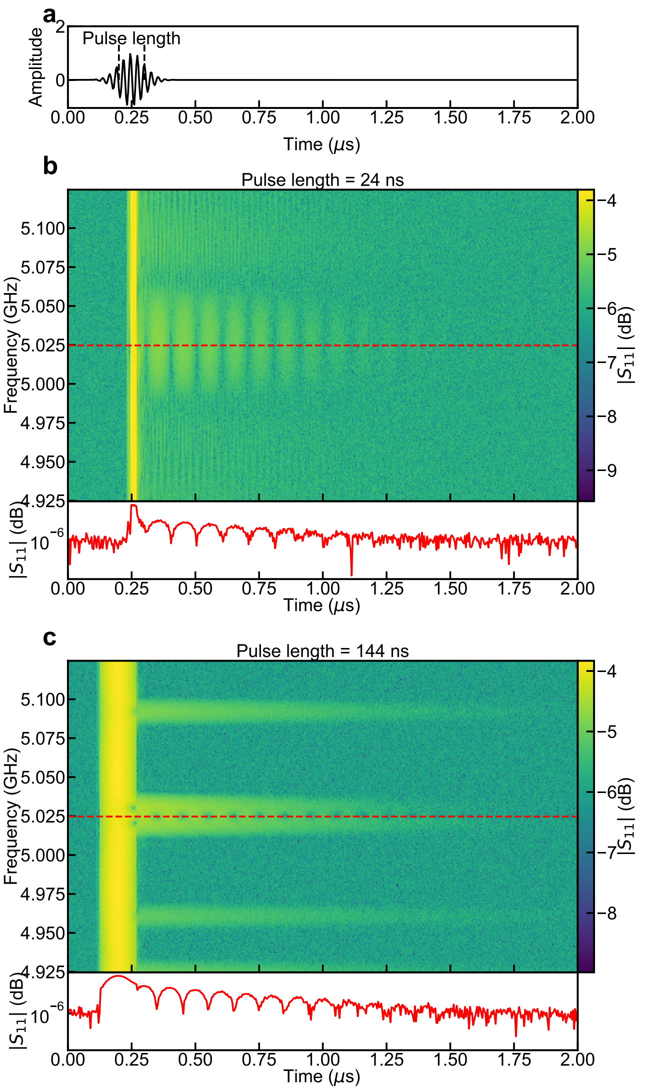

Supplementary Figure S19. **Calibration of the time resolved measurements. a.** Gaussian pulse sent for the calibration measurement. **b.** (Top) Time-resolved measurement as a function of the frequency of the applied pulse, for a pulse length of 24 ns on a CCA with 26 cavities in the configuration $J_2/J_1 = 1.22$. (Bottom) Line-cut at the position of the red dashed line (Top). **c.** (Top) Time-resolved measurement as a function of the frequency of the applied pulse, for a pulse length of 144 ns on a CCA with 26 cavities in the configuration $J_2/J_1 = 1.22$. (Bottom) Line-cut at the position of the red dashed line (Top).

[10] A. Eichler and O. Zilberberg, *Classical and Quantum Parametric Phenomena* (Oxford University Press, Oxford, 2023).

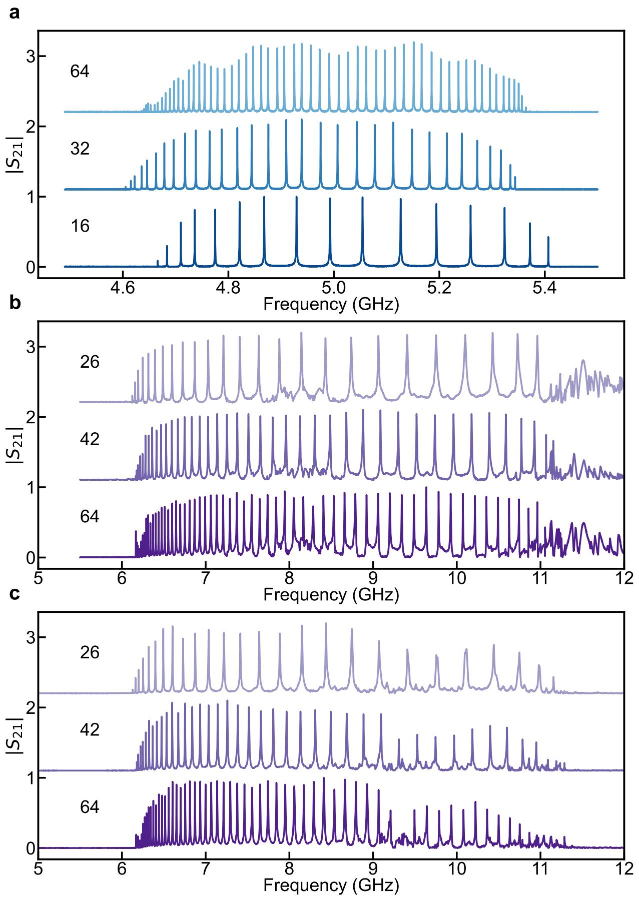

Supplementary Figure S20. **Extra spectra of CCAs with $M = 1$ a.** Transmission measurements, $|S_{21}|$, from a uniform rectangular CCA ($M = 1$) with $N = 16, 32, 64$ and $J/2\pi = 180\,\text{MHz}$. **b** Transmission measurements, $|S_{21}|$, from a uniform hexagonal CCA ($M = 1$) with $N = 26, 42, 64$ and $J/2\pi = 1200\,\text{MHz}$. Partially reproduced in Fig. 2**c**. **b.** Same device measured in a different cooldown, with different sample packaging.

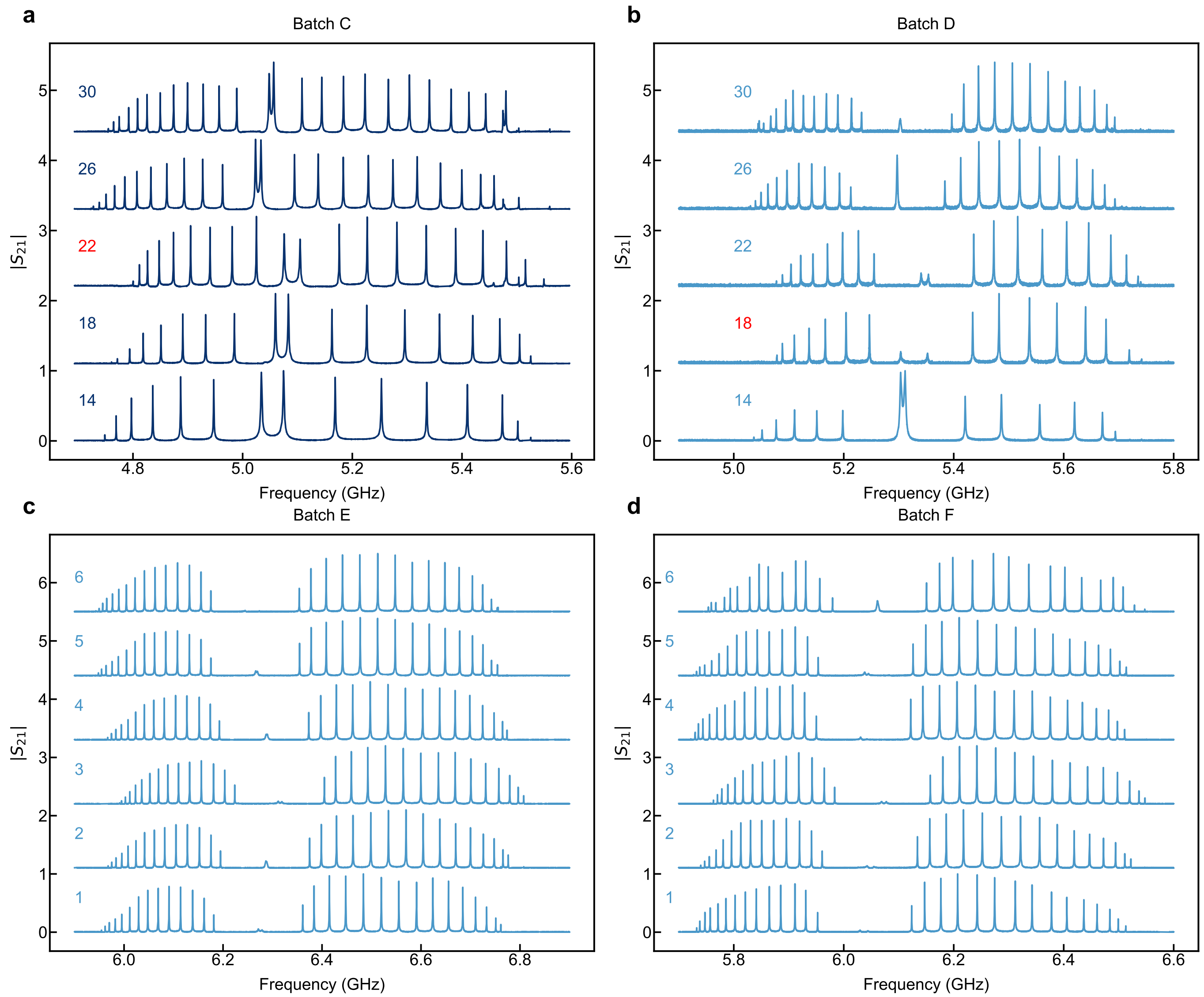

Supplementary Figure S21. **Spectrum of SSH CCA used in Fig. 4 a.** Amplitude of the CCA transmission, $|S_{21}|$, of devices of batch C. **b.** Amplitude of the CCA transmission, $|S_{21}|$, of devices of batch D. **c.** Amplitude of the CCA transmission, $|S_{21}|$, of devices of batch E. **d.** Amplitude of the CCA transmission, $|S_{21}|$, of devices of batch F. The strong and light blue colors highlight devices in the configuration $J_2/J_1 = 1.22$ and $J_2/J_1 = 1.57$ configurations, respectively. The CCAs in batches C and D are fabricated with a different number of resonators ($N = 14, 18, 22, 26, 30$). Batch E and F consist of 6 repetitions of identical CCA with $N = 32$. The red labels highlight spectra of CCAs presenting strong local disorder identified according to time-resolved measurements (see Sec. IV of the main text).

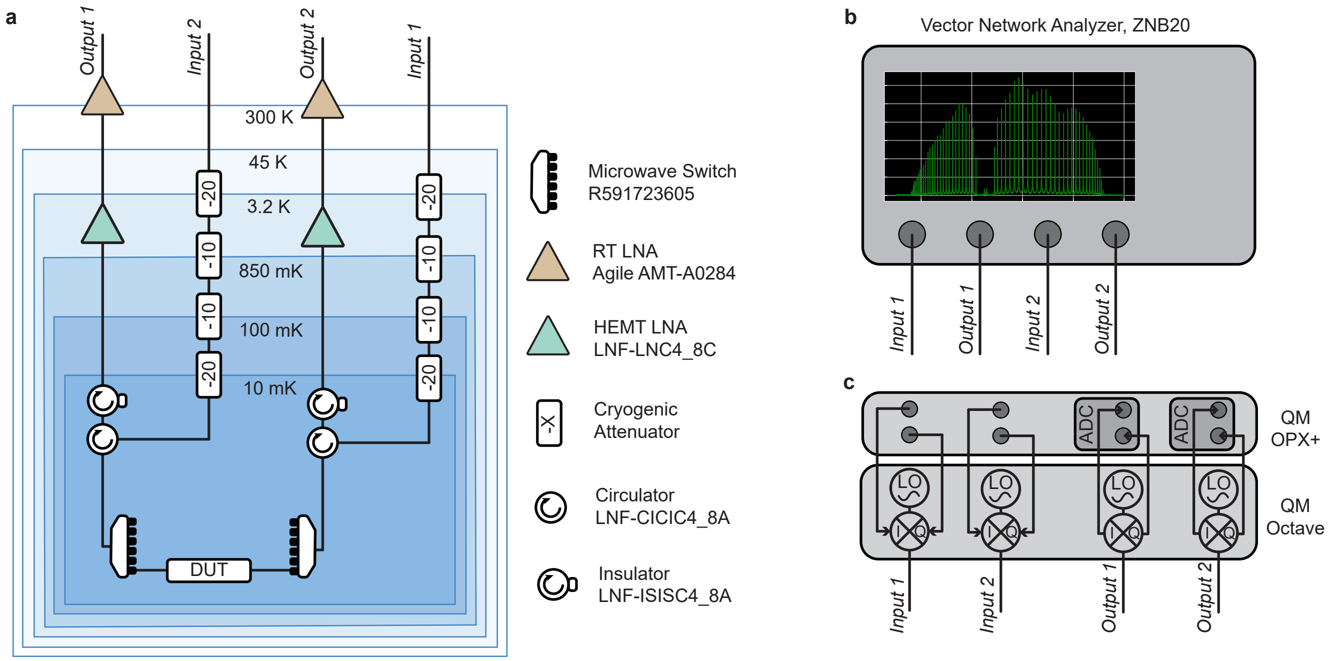

Supplementary Figure S22. **Schematic of the measurement setup. a.** Cabling of the cryogenic setup. The input lines are attenuated with 60 dB cryogenic attenuators distributed along the different stages of the cryostat. **b.** Cabling of the room-temperature Vector Network Analyzer (VNA). The input/output lines correspond to the one of panel **a**. **c.** Cabling of the room-temperature time-domain setup. The input/output lines correspond to the one of panel **a**.
